# Salt-Inducible Kinase 1 is a potential therapeutic target in Desmoplastic Small Round Cell Tumor

**DOI:** 10.1038/s41389-022-00395-6

**Published:** 2022-04-20

**Authors:** Alifiani Bonita Hartono, Hong-Jun Kang, Lawrence Shi, Whitney Phipps, Nathan Ungerleider, Alexandra Giardina, WeiPing Chen, Lee Spraggon, Romel Somwar, Krzysztof Moroz, David H. Drewry, Matthew E. Burow, Erik Flemington, Marc Ladanyi, Sean Bong Lee

**Affiliations:** 1 Tulane University School of Medicine, Department of Pathology and Laboratory Medicine, New Orleans, LA USA; 2 Genomics Core, National Institute of Diabetes and Digestive and Kidney Diseases, Maryland, USA; 3 Department of Pathology, Memorial Sloan Kettering Cancer Center, New York, NY USA; 4 University of North Carolina, Eshelman School of Pharmacy, Chapel Hill, NC USA; 5 Department of Medicine, New Orleans, LA USA

**Keywords:** Paediatric cancer, Oncogenes

## Abstract

Desmoplastic Small Round Cell Tumor (DSRCT) is a rare and aggressive malignant cancer caused by a chromosomal translocation t(11;22)(p13;q12) that produces an oncogenic transcription factor, EWSR1-WT1. EWSR1-WT1 is essential for the initiation and progression of DSRCT. However, the precise mechanism by which EWSR1-WT1 drives DSRCT oncogenesis remains unresolved. Through our integrative gene expression analysis, we identified Salt Inducible Kinase 1 (SIK1) as a direct target of EWSR1-WT1. SIK1 as a member of the AMPK related kinase is involved in many biological processes. We showed that depletion of SIK1 causes inhibition of tumor cell growth, similar to the growth inhibition observed when EWSR1-WT1 is depleted. We further showed that silencing SIK1 leads to cessation of DNA replication in DSRCT cells and inhibition of tumor growth in vivo. Lastly, combined inhibition of SIK1 and CHEK1with small molecule inhibitors, YKL-05-099 and prexasertib, respectively, showed enhanced cytotoxicity in DSRCT cells compared to inhibition of either kinases alone. This work identified SIK1 as a new potential therapeutic target in DSRCT and the efficacy of SIK1 inhibition may be improved when combined with other intervention strategies.

## Introduction

Desmoplastic small round cell tumor (DSRCT) is an aggressive adolescent tumor arising in the serosal surface of the abdominal or pelvic cavity. Tumors form multiple nests of highly malignant cells surrounded by dense desmoplastic stroma and typically metastasize to the liver and spleen [1]. Despite resection of the tumors and aggressive chemo and radiation therapy, the overall survival rate five years after initial diagnosis is dismal (5–30%) [2], emphasizing the urgent need for more efficacious therapy.

DSRCT is driven by an oncogenic fusion protein generated by a chromosomal translocation t(11;22)(p13;q12) that juxaposes the promoter and the N-terminal exons of *EWSR1 (Ewing sarcoma breakpoint region 1)* to the last 3 exons of *WT1* (*Wilms Tumor 1*) [3]. The N-terminal exons of *EWSR1* encode a disordered domain, which imparts a potent transcriptional activation activity to the fusion protein [4, 5]. WT1 encodes a C2H2 zinc finger transcription factor and the EWSR1-WT1 fusion protein contains the last 3 zinc fingers of WT1. An alternative splicing that inserts 3 amino acids Lys, Thr, and Ser (KTS) between the zinc finger 3 and 4 in WT1 [6] also occurs in the fusion transcript, generating two different isoforms: EWSR1-WT1(-KTS) and EWSR1-WT1(+KTS) [herein designated E-KTS and E+KTS], that differ in their DNA binding and oncogenic properties [7, 8]. E-KTS recognizes a 9-bp GC-rich sequences whereas E+KTS recognizes a 6-bp GAA-repeats [8–10]. E-KTS can transform NIH3T3 cells while E+KTS cannot [7] and overexpression of E-KTS, but not E+KTS, causes oncogenic stress that leads to cell cycle arrest in both primary cells and in mice [11]. Additionally, E-KTS drives neural gene expression in mouse fibroblasts and induces more transcriptional changes than the E+KTS [11]. Although several direct target genes of both isoforms have been discovered [12], the exact mechanism by which EWSR1-WT1 fusion drives tumorigenesis in DSRCT is largely unknown. This has hampered the development of targeted therapies in DSRCT. In this work, we sought to identify direct EWSR1-WT1 target genes that might serve as actionable therapeutic targets in DSRCT.

## Results

### Identification of SIK1 as a direct target of EWSR1-WT1

To identify new therapeutic targets in DSRCT, we performed integrative gene expression analysis using DSRCT cells and primary tumors. First, we depleted *EWSR1-WT1* in JN-DSRCT-1 (JN) cells using shRNA directed against the 3′UTR of *WT1*. Since wildtype WT1 is not expressed in DSRCT cells ([13] and Fig. S1A), the shRNA is expected to specifically target EWSR1-WT1. Microarray expression profiling of JN cells with EWSR1-WT1 depletion compared to shRNA controls detected changes in more than 3,500 transcripts. We then cross-referenced these transcripts with our previously identified primary DSRCT-enriched data set, where we performed a pair-wise gene expression analysis of 28 primary DSRCT with other sarcomas: 28 Ewing sarcoma (ES), 23 alveolar rhabdomyosarcoma (ARMS), 46 synovial sarcoma (SS) or 12 alveolar soft part sarcoma (ASPS) [11]. The integrated gene expression analysis identified 201 DSRCT-enriched and 74 DSRCT-repressed genes (Supplement Table S1). Gene ontology (GO) analysis revealed protein kinases and transcription factors as the two most enriched classes of genes upregulated in DSRCT.

**Fig. 1:**
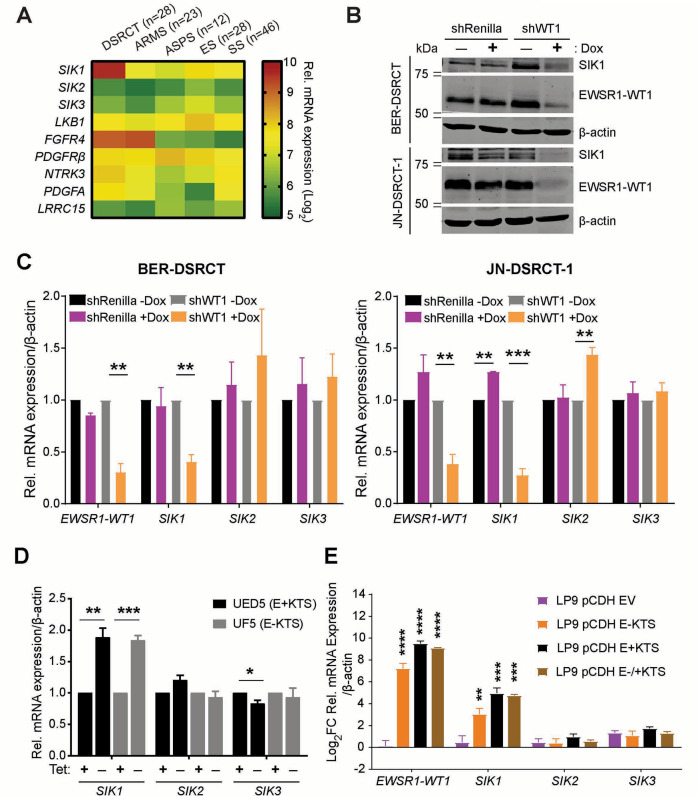
SIK1 expression is regulated by EWSR1-WT1. **A** Heatmap of relative transcript levels of *SIK*-family and other kinases in DSRCT, ARMS, ASPS, ES, SS primary tumors based on Affymetrix U133A expression array data. **B** EWSR1-WT1 and SIK1 protein levels in dox-inducible shRenilla or shWT1 JN and BER-DSRCT stable cell lines. **C** Relative mRNA expressions of *EWSR1-WT1*, *SIK1*, *SIK2*, and *SIK3* in JN and BER-DSRCT shRenilla or shWT1 inducible cell lines with or without Dox treatment. **D** Relative mRNA expression of *SIK1*, *SIK2*, and *SIK3* in TET-off inducible UF5 (E - KTS) and UED5 (E+KTS) cells (*p < 0.05, **p < 0.01, ***p < 0.001, mean ± SEM, student *t*-test). **E** Relative mRNA expressions of *EWSR1-WT1*, *SIK1*, *SIK2*, and *SIK3* in LP9 cells that have been transduced with a control lentivirus (empty vector (EV)) or with lentiviruses expressing E-KTS, E+KTS, or both isoforms (E−/+KTS). mRNA expression in transduced cells were compared to the non-transduced LP9 cells. (**p < 0.01, ***p < 0.001, ****p < 0.0001, mean ± SEM, student *t*-test).

Among the DSRCT-enriched kinases, we focused on Salt-Inducible Kinase 1 (SIK1) since it has not been studied in DSRCT. SIK1 has two other members in its family, SIK2 and SIK3, and as a member of AMPK related kinase, SIK1 can be activated by LKB1 [14]. Thus, we examined the expression levels of *SIK* and *LKB1* in the microarray expression data of primary DSRCT tumors. Expression of *SIK1* was the highest in DSRCT compared to other sarcomas (Fig. 1A), while expression levels of *SIK2*, *SIK3* and *LKB1* in DSRCT were similar to other sarcomas. As expected, previously identified EWSR1-WT1 targets such as *FGFR4* [15], *PDGFA* [16], *PDGFRB* [10] and *NTRK3* [13] were also enriched in DSRCT.

One of the intriguing features about SIK1 is its purported duplication in humans, resulting in a second SIK1 kinase termed SIK1B, which is not present in rodents and lower vertebrates [17]. BLAST alignment of ~19 kb human *SIK1* and *SIK1B* genomic regions encompassing the proximal promoter, exons/introns and 3’UTR showed only 18 bp differences (Supplement Table S2). Notably, there are only 2 bp alterations between the coding regions of *SIK1* and *SIK1B*, a non-synonymous single-nucleotide polymorphism (SNP) in exon 13 leading to Ala615 (GCC) in *SIK1* to Val615 (GTC) in *SIK1B* and a synonymous SNP (Pro616, CCC to CCT in *SIK1* and *SIK1B* respectively). These as well as 5 additional SNPs in the 3′ UTR can be used to differentiate between *SIK1* and *SIK1B* transcripts. However, recent completion of the missing gaps in the human genome suggested that the duplication of chromosome 21 segment containing *SIK1* arose erroneously due to false duplications in either GRCh37 or GRCh38 [18, 19]. Therefore, we decided to carefully examine the *SIK1/SIK1B* loci copy number variation (CNV) as well as direct sequencing of SIK1/SIK1B exon 13 genomic DNA. For CNV analysis, we performed qPCR analysis with genomic DNAs isolated from control and DSRCT cells using TaqMan probes against *SIK1* or a control gene *RPP30* on chromosome 10. Our results showed that *SIK1/SIK1B* loci are not duplicated compared to a nonduplicated *RPP30* control (Supplement Fig. S1B). Furthermore, direct sequencing of exon 13 genomic DNA from LP9, JN and BER-DSRCT (BER) cells showed only Val615 SNPs, which further showed that *SIK1* is not duplicated (Supplement Fig. S1C). Presence of only Val615 (GTC) variant was confirmed by direct sequencing of exon 13 and the 3′ UTR regions of *SIK1* transcripts in JN and BER cells (Supplement Fig. 1D, E). Together, these results provide clear evidence that *SIK1* is not duplicated and that Ala615 and Val615 variants likely represent SNPs in *SIK1*.

### EWSR1-WT1 directly regulates SIK1 expression in DSRCT

To determine whether *SIK1* is directly regulated by EWSR1-WT1, we established doxycycline (dox)-inducible shRNA stable cell lines against EWSR1-WT1 (3′ UTR of *WT1*) or control (shRenilla) in JN and BER cells. Treating the shWT1 JN and BER stable cells with dox for 5 or 3 days, respectively, resulted in marked depletion of EWSR1-WT1 as well as SIK1 proteins (Fig. 1B). Through qRT-PCR, we determined that only *SIK1* expression, but not *SIK2* or *SIK3*, was affected by depletion of EWSR1-WT1 (Fig. 1C). Conversely, when EWSR1-WT1 was overexpressed in an inducible U2OS cell lines, UF5 (E–KTS) and UED5 (E+KTS), only *SIK1* expression, but not *SIK2* or *SIK3*, was induced (Fig. 1D). We observed similar findings in LP9 cells, a normal human mesothelial cell which represents one of the potential DSRCT tumor cells of origin (Fig. 1E). Notably, both isoforms were capable of inducing expression of *SIK1* in U2OS and LP9 cells. Collectively, these data showed that EWSR1-WT1 regulates only *SIK1* among the SIK-family kinases in DSRCT and human mesothelial cells.

**Fig. 2:**
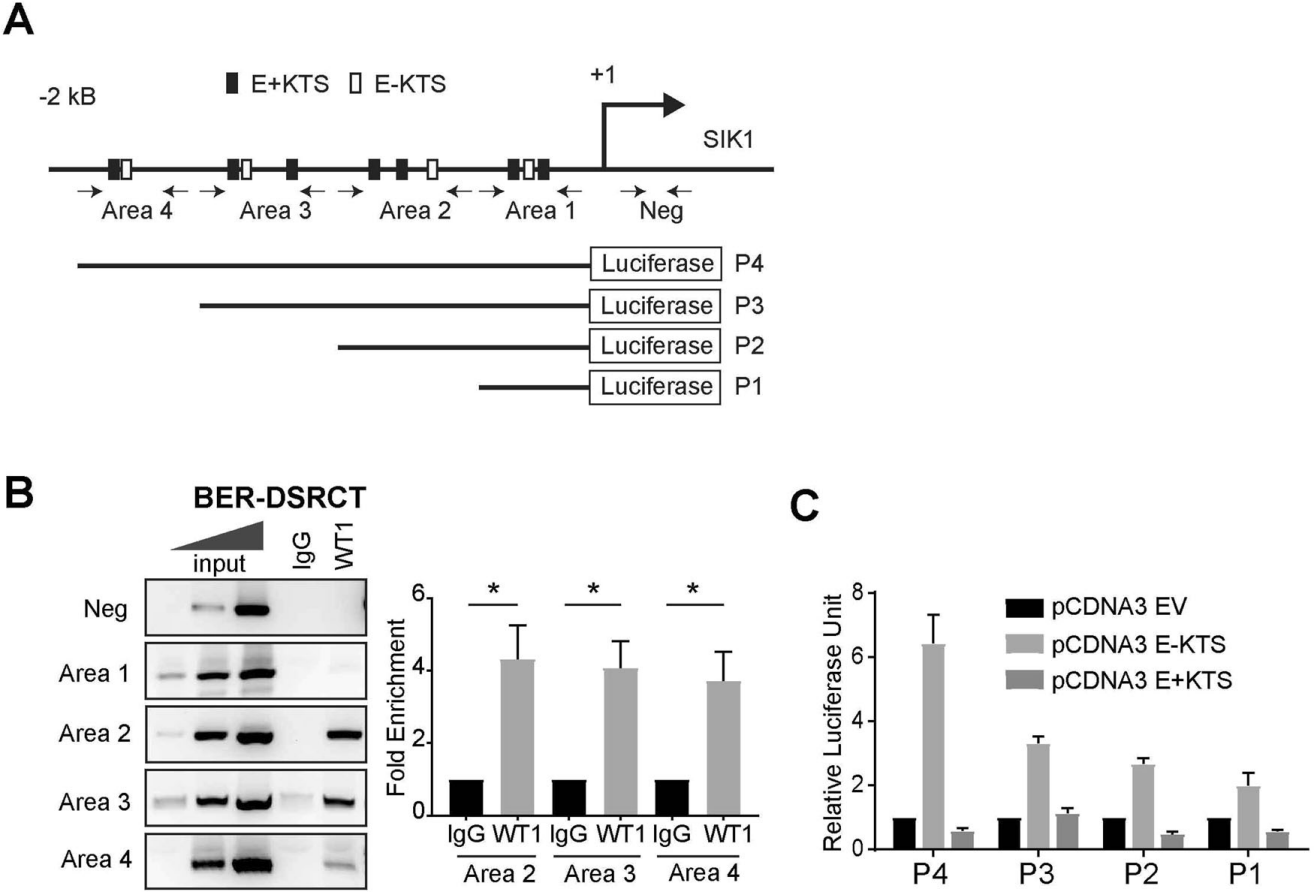
EWSR1-WT1 directly activates SIK1 expression. **A** Schematic showing putative E-KTS and E+KTS binding sites in SIK1 2kB proximal promoter and P1-P4 luciferase reporter constructs. **B** ChIP analysis of SIK1 promoter with IgG or WT1 C-term antibody in BER-DSRCT cells. Areas 1–4 and negative regions were PCR-amplified with the indicated primers (arrows in C). Bands intensities were quantified using ImageJ and normalized to input (*n* = 3, *p < 0.05, mean ± SEM, student *t*-test, lower panel). **C** EWSR1-WT1 directly activates SIK1 promoter. U2OS cells were transfected with pCDNA3-E-KTS, E+KTS or pCDNA3-Empty Vector and P1-P4 reporter constructs. Renilla Luciferase plasmid was used to normalize for transfection efficiency. Relative luciferase activities were calculated relative to pCDNA3-empty vector from 4 independent experiments (mean ± SEM).

We next examined the 2 kb *SIK1* proximal promoter region and found multiple E-KTS and E+KTS binding sites (Fig. 2A). Chromatin Immunoprecipitation (ChIP) analysis of JN and BER cells showed that EWSR1-WT1 bound specific regions in the *SIK1* promoter (Fig. 2B and Supplement Fig. S2A). Sequencing analysis of the ChIP-PCR products revealed the presence of SNPs in the *SIK1* promoter regions (Supplement Fig. S2B). To determine whether *SIK1* is a direct transcriptional target of EWSR1-WT1, we cloned a 2 kb proximal promoter region (P4) into a promoterless luciferase plasmid pGL3Basic and generated serial deletion promoter-reporter constructs. Transfection of P4 and E-KTS expression constructs resulted in a robust expression of luciferase reporter (Fig. 2C). Deletion of Area 4 resulted in about 50% decrease in luciferase expression and deletions of Areas 3 and 2 resulted in further but modest reductions, demonstrating that Area 4 contains the strongest E-KTS regulatory elements. Although SIK1 expression was induced by E+KTS in UED5 and LP9 cells, expression of E+KTS did not activate the reporter expression, suggesting that the regulatory elements for E+KTS reside outside of the 2 kb proximal promoter region. These results demonstrate that EWSR1-WT1 directly regulate SIK1 expression in DSRCT.

### SIK1 is essential for DSRCT cell growth

**Fig. 3:**
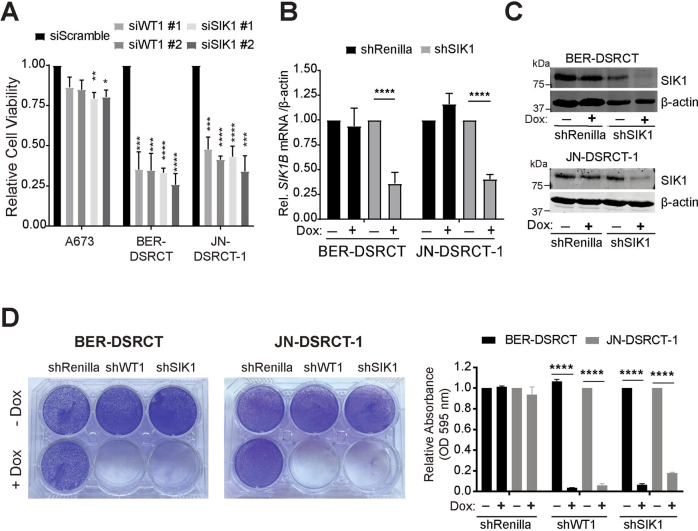
SIK1 is essential for DSRCT cell growth. **A** A673, BER-DSRCT, and JN-DSRCT-1 cells were transfected with two independent siWT1, siSIK1, or siScramble (control). Cell viability was measured 3 days after transfection. (*p < 0.05**p < 0.01, ***p < 0.001, ****p < 0.0001, mean ± SEM, student *t*-test). **B** Relative mRNA expression of SIK1 after 3 days of doxycycline treatment (****p < 0.0001, mean ± SEM, student *t*-test). **C** Western blot analyses showing SIK1 is effec*t*ively reduced in Dox-treated shSIK1 JN and BER-DSRCT cells compared to untreated or control cells. **D** Colony formation assay of dox-inducible shRenilla, shWT1, or shSIK1 DSRCT cell lines after 14 days of dox treatment (****p < 0.0001, mean ± SEM, student *t*-test). The right panel shows quantification of the stained cells in the colony formation assay.

To determine the role of SIK1 in DSRCT, we depleted SIK1 in JN, BER, and A673 cells, an Ewing Sarcoma cell line, with two independent siRNAs against EWSR1-WT1, SIK1, or a scramble siRNA (Fig. 3A and Supplement Fig. S3). A673 cell viability was not affected when transfected with siWT1 and only modestly reduced when SIK1 was depleted. However, JN and BER cell viability decreased by more than 50% when EWSR1-WT1 or SIK1 was depleted, suggesting that SIK1 is essential for DSRCT cells. We next generated dox-inducible shSIK1 stable JN and BER cells. Addition of dox effectively reduced SIK1 transcript (Fig. 3B) and protein (Fig. 3C) levels in BER and JN cells. Importantly, silencing SIK1 inhibited DSRCT cell growth similar to EWSR1-WT1 depletion (Fig. 3D).

**Fig. 4:**
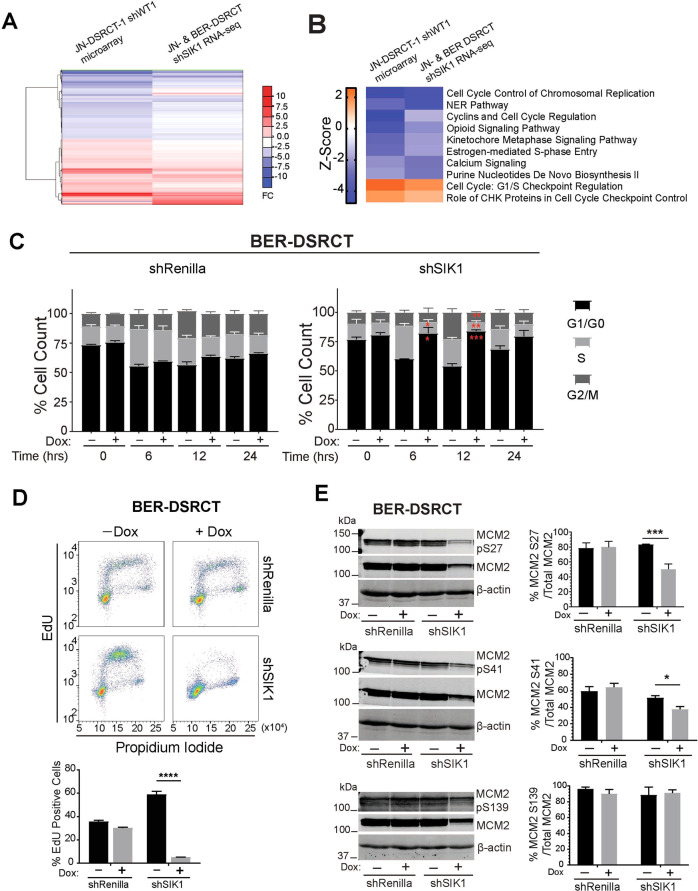
SIK1 is essential for DNA replication in DSRCT cells. **A** Heatmap of commonly regulated genes following EWSR1-WT1 or SIK1 depletion in DSRCT cells. **B** Top common pathways that were altered in IPA (Qiagen) following EWSR1-WT1 or SIK1 depletion in DSRCT cells. **C** Cell cycle analysis of G1-arrested shRenilla or shSIK1 DSRCT cell lines with or without Dox treatment following release to S phase. Quantification from three independent experiments are shown. **D** EdU incorporation analysis of dox-inducible shRenilla and shSIK1 BER-DSRCT stable cell lines. A representative flow cytometry data from three independent experiments are shown in the upper panel. Percent EdU+ cells (lower panel) were calculated from three independent experiments (***p < 0.001, ****p < 0.0001, mean ± SEM, student *t*-test). **E** SIK1 deple*t*ion reduces MCM2 phosphorylation at S27 and S41 sites. Western blot with specific phospho-specific antibodies to MCM2 S27, S41, S139, and MCM2. Phosphorylation signal intensity was normalized to total MCM2 levels (*n* = 3, *p < 0.05, ***p < 0.001 mean ± SEM, student *t*-test).

To identify potential mechanisms underlying SIK1-mediated growth inhibition, next generation sequencing of transcripts (RNA-seq) was performed in SIK1-depleted JN and BER cells. We cross-referenced this expression data with the microarray data of EWSR1-WT1 depleted JN-DSRCT-1 cells (Fig. 4A, Supplement Table S3). Ingenuity Pathway Analysis (IPA) of commonly altered transcripts in both data sets revealed “cell cycle control of chromosomal replication” as the top pathway that was affected when either EWSR1-WT1 or SIK1 was depleted (Fig. 4B and Supplement Table S4). To explore the role of SIK1 in regulating the cell cycle in DSRCT cells, JN-shRenilla, JN-shSIK1, BER-shRenilla, and BER-SIK1 cells were synchronized at G1 with or without dox, cells were released into S-phase and their cell cycle profiles were analyzed by flow cytometry at various times. Depletion of SIK1 in JN and BER cells prevented entry into S-phase while the untreated (-dox) or control JN and BER cells with or without dox were able to progress normally through the cell cycle (Fig. 4C and Supplement Fig. S4A). Concomitantly, Cyclin E expression, which accumulated during late G1/S and declined during S and G2/M phases in untreated cells, remained constant across all time points when SIK1 was depleted (Supplement Fig. S4B).

A recent study has shown that SIK1 is involved in DNA replication by regulating the MCM DNA helicase [20]. Thus, we measured DNA replication in DSRCT cells with or without SIK1 depletion. Synchronously G1-arrested BER and JN cells were released to enter S-phase, pulsed for one hour with EdU, and collected for flow cytometry analysis. SIK1 depletion resulted in a near complete inhibition of DNA replication in BER cells (Fig. 4D) and significant decrease in JN cells (Supplement Fig. S4C). shRenilla control cells (with or without dox) or untreated (-dox) shSIK1 cells did not show any effects on DNA replication. Next, we examined the MCM2 phosphorylation at S27, S41, and S139, which are reported SIK1 phosphorylation sites [20], in DSRCT cells with or without SIK1 depletion using phospho-specific antibodies. Phosphorylation at S27 and S41 was significantly reduced when SIK1 was depleted compared to controls, but not S139 (Fig. 4E and Supplement Fig. S4D). Similar findings were observed with a pharmacological inhibition of SIK1 with a pan-SIK inhibitor YKL-05-099, which inhibits all three SIK-family kinases [21] (Supplement Fig. S5). Notably, total MCM2 levels were markedly reduced when SIK1 was depleted (Fig. 4E), raising the possibility that SIK1 may also regulate MCM2 stability or expression in DSRCT cells.

### Inhibition of SIK1 is effective in reducing xenograft growth

**Fig. 5:**
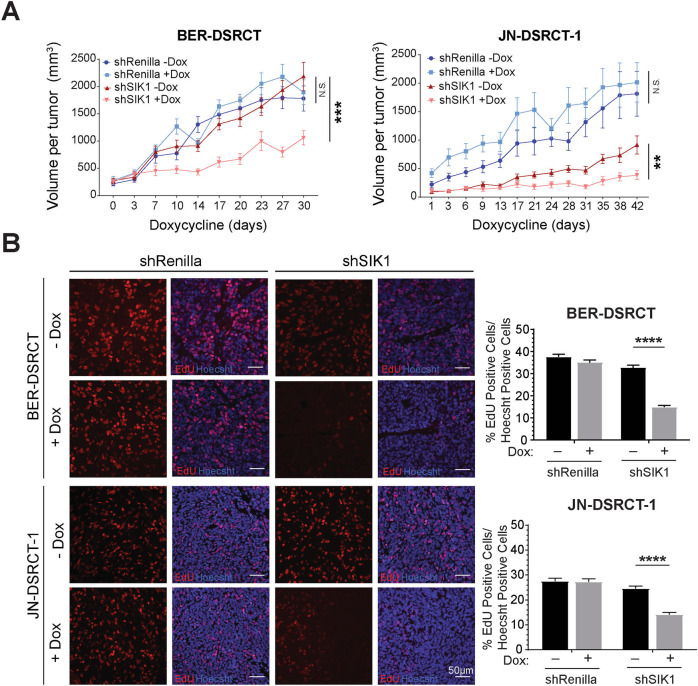
SIK1 is essential for DSRCT xenograft growth. **A** Dox-inducible shRenilla and shSIK1 JN- and BER-DSRCT cells were injected subcutaneously into bilateral flanks of NSG mice, randomized into two groups (*n* = 5/group) and one cohort received doxycycline containing drinking water while the other received normal water. Tumors were measured every 3 days using digital calipers. A total of 10 tumors/group were measured, **p < 0.01, ***p < 0.001, mean ± SEM, Two-way ANOVA. **B** Representative images of EdU+ cells in xenograft tumors. Tumors sections were photographed under immunofluorescence microscope and three random fields per tumor section were quantified for EdU+ cells and Hoecsht positive cells (*n* = 10, ****p < 0.0001, mean ± SEM, student *t*-test).

To examine the effects of targeting SIK1 in DSRCT tumor growth in vivo, JN- and BER-shSIK1 and shRenilla stable cells were injected into immune-deficient NOD.SCID/IL2Rγ-null (NSG) mice. When tumors became palpable, shRNA expression was induced by adding doxycycline in the drinking water and tumor growth was measured every 3 days. No discernable body weight changes were noted in any of the mice during the entire study. Consistent with the in vitro studies, SIK1 depletion resulted in a significant inhibition of tumor growth of both JN- and BER-shSIK1 xenografts but not in the untreated (except JN-shSIK1) nor in control xenografts (Fig. 5A). Tumor growth was noticeably slow in JN-shSIK1 cells even in the absence of dox, which might be due to leaky expression of shSIK1 in these cells (Supplement Fig. S6) or to some other unknown reasons. To examine the effects of SIK1 depletion on xenograft tumor DNA replication, EdU was injected intraperitoneally before mice were euthanized. Tumors were harvested and stained for EdU-incorporated DNA. Consistent with our in vitro findings, SIK1 depletion resulted in significantly fewer EdU+ cells in xenografts compared to controls (Fig. 5B), demonstrating a requirement for SIK1 in replicating tumor cell DNA in vivo.

### Combined inhibition of SIK1 and CHEK1 shows enhanced efficacy in DSRCT cells

**Fig. 6:**
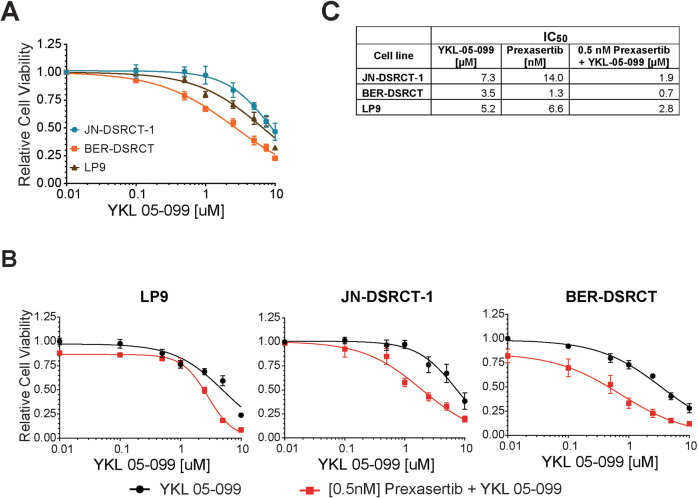
Combined inhibition of SIK1 and CHEK1 shows enhanced efficacy in DSRCT cells. Dose-response curves of JN-DSRCT-1, BER-DSRCT, and LP9 cells treated with **A** YKL-05–099 alone or **B** in combination with 0.5 nM prexasertib and increasing doses of YKL-05–099. DSRCT cell viability values were compared to LP9 cell viability at each dose (*p < 0.05, **p < 0.01, ***p < 0.001, mean ± SEM, student *t*-test). **C** IC50 values for YKL-05–099, prexasertib, or combination treatement (0.5 nM prexasertib with increasing doses of YKL-05–099).

Currently, there is no specific small molecule inhibitor against SIK1. Therefore, a pan-SIK inhibitor, YKL-05-099, was tested. JN and BER, along with LP9 cells, were grown in the presence of increasing doses of YKL-05-099 and cell viability was measured. Notably, BER cells were significantly more sensitive to YKL-05-099 with IC50 at 3.5 µM (Fig. 6A, C). However, JN cells did not show any increased cytotoxicity to the inhibitor compared to LP9 cells. We currently do not understand the reason underlying this discrepancy.

Prexasertib is an inhibitor of Checkpoint Kinase 1 (CHEK1) that was recently shown to be effective in pediatric tumor PDX models, including DSRCT [22]. Since CHEK1 is activated during DNA replication stress or DNA damage [23], we surmised that combined inhibition of SIK1 and CHEK1 could be effective in DSRCT. We first determined the effective dose of prexasertib in JN and BER cells and found that similar to YKL-05-099, BER cells were more sensitive to prexasertib than JN or LP9 cells (Supplement Fig. S7). Importantly, at 0.5 nM prexasertib, JN and LP9 cells were not affected and BER cells showed only modest loss of cell viability. Remarkably, when 0.5 nM prexasertib was combined with 1 µM of YKL-05-099, nearly 50% of JN cells showed cytotoxicity (Fig. 6B), whereas more than 80% of JN cells were viable at 1 µM of YKL-05-099 alone (Fig. 6A). BER cells also showed significant increase in cytotoxicity when co-treated with the same doses of both inhibitors than either drug alone. In contrast, LP9 cells were not as sensitive to the combined treatments at similar doses, only demonstrating greater than 50% loss at 2.5 µM or higher doses of YKL-05-099 and 0.5 nM prexasertib. These results suggest that targeting SIK1 may augment the cytotoxic effects of prexasertib on tumor growth in DSRCT.

## Discussion

Poor prognosis of DSRCT patients despite undergoing aggressive multimodal therapy highlights the urgent need for a more DSRCT-specific therapy. DSRCT tumorigenesis is driven by the expression of EWSR1-WT1 fusion protein and recent whole genome sequencing of 10 DSRCT primary tumors detected no recurrent secondary oncogenic genetic alterations [24]. Targeting fusion transcription factor proteins such as EWSR1-WT1 with small molecular inhibitors has proven difficult [25]. Therefore, identifying EWSR1-WT1 regulated genes and pathways that are essential for DSRCT viability could uncover new therapeutic targets and suggest potential combinational drug treatment with the current standard of care. The integrative expression analysis to cross-reference gene expression changes induced by EWSR1-WT1 depletion in DSRCT cells to the highly enriched transcripts in primary DSRCT has identified SIK1 as a potential novel target. A previous RNA-seq analysis of transient knockdown of EWSR1-WT1 in JN and BER-DSRCT cell lines also identified SIK1 as one of the top EWSR1-WT1-regulated genes [26]. Proximal 2 kb SIK1 promoter region harbors multiple E-KTS and E+KTS binding sites, but only E-KTS is able to activate this promoter, which suggests that E+KTS regulatory elements reside outside of this region.

A region in chromosome 21 encompasssing *SIK1* and the neighboring genes was initially thought to be duplicated [17]. However, our CNV and genomic DNA sequencing analyses provide clear evidence against *SIK1* duplication. The *SIK1B (Val615)* variant differs from *SIK1* by only two C- > T changes in the coding region that results in a nonsynonomous (Ala615 to Val) and a synonomous (Pro616) changes near the C-terminal region. While the functional consequences of Ala615 to Val alteration is unknown, Hansen et al. [27] found that in epilepsy patients with SIK1 missense mutations near the C-terminal region, the kinases were able to shuttle between the nucleus and cytoplasm similarly to wild type SIK1 under PKA stimulation. Importantly, these SIK1 mutants still retained normal kinase function/activity, suggesting that the Val615 variant is likely to be as functional as the Ala615. Phosphorylation of SIK1 at Thr182 residue by LKB1 activates and shuttle SIK1 to the nucleus [14]. In DSRCT cells, SIK1-Val615 variant could be found in both cytoplasm and nucleus, and Thr182 phosphorylation was detected only in the nuclear extracts (data not shown), suggesting that SIK1-Val615 in DSRCT is regulated similarly to SIK1-Ala615.

The current study clearly demonstrated that SIK1 is an oncogenic kinase in DSRCT based on its high expression in primary tumors and as a direct target of EWSR1-WT1. Importantly, depletion of SIK1 leads to rapid growth arrest at G1/S phase in DSRCT cells and xenografts, likely due to reduced phosphorylation and activation of MCM2. Although SIK1 has previously been shown to be a tumor suppressor due to its role in promoting p53-dependent anoikis in breast cancer [28] and mediating tumor-suppressor function of LKB1 in lung cancer [29], the oncogenic role of SIK1 in DSRCT is in line with recent observations that SIK1 is able to promote cell growth in oxygen-glucose deprived mouse neuro-endothelial cells [30] and in down-regulating the p53 apoptosis pathway in medulloblastoma [31]. The opposing nature of SIK1 in promoting or suppressing cell viability illustrates that the functions of SIK1 can be tumor cell-specific.

One of the top common pathways affected by the depletion of EWSR1-WT1 or SIK1 in JN and BER cells was cell cycle regulation, which led us to uncover the mechanisms underlying cell cycle arrest. A recent study has implicated SIK1 in activating the MCM DNA helicase complex through phosphorylation of the N-terminal domain of MCM2 in an in vitro kinase assay [20]. Phosphorylation of MCM proteins is critical in regulating DNA replication and cell cycle progression, and aberrant phosphorylation can lead to genomic instability and the development of cancers [32]. Our novel findings that depletion of SIK1 in JN and BER cells results in an inhibition of DNA replication and decreased phosphorylation of MCM2 revealed for the first time a critical role of SIK1 in DSRCT cell growth, highlighting SIK1 as a potential therapeutic target in DSRCT. Other kinases such as Cdc7, an S-phase promoting kinase, and CDK2 are capable of phosphorylating MCM2 [33, 34] and both kinases are expressed in DSRCT. However, our studies clearly demonstrate that loss of SIK1 cannot be compensated by other kinases in DSRCT cells. Remarkably, SIK1 appears to regulate the stability and/or expression of MCM2 protein as depletion of SIK1 in DSRCT cells led to marked reduction in MCM2 protein level. It would be interesting to determine whether SIK1 regulates both the stability and activity of MCM2 in DSRCT and in other cell types.

A specific chemical inhibitor of SIK1 is currently unavailable, and thus, a pan-SIK inhibitor, YKL-05-099, was tested on DSRCT cells. Out of the two DSRCT cell lines, BER cells were more sensitive to YKL-05-099 than JN cells, even though the expression levels of SIK1 are similar in the two cell lines. We currently do not understand this discrepancy but given that SIK1 depletion via shRNA was effective in both cells, a more potent and specific SIK1 inhibitor will likely show improved efficacy in both DSRCT cells.

Currently, a clinical trial to evaluate the efficacy of the CHEK1 inhibitor prexasertib in DSRCT patients is ongoing (NCT04095221). Since silencing SIK1 in DSRCT cells causes a G1/S cell cycle arrest, we hypothesized that combined SIK1 and CHEK1 inhibition could potentially be more effective in DSRCT cells than either treatment alone. Indeed, there was an enhanced cytotoxicity to low doses of prexasertib and YKL-05-099 in both JN and BER cells than either drug alone, implicating a combined inhibition as a potential therapy in DSRCT. However, a more potent and specific SIK1 inhibitor will need to be developed before the combined treatment can be considered.

## Materials and methods

### Cell lines and reagents

JN-DSRCT-1 (JN) [35] and BER-DSRCT (BER) cells [36] were previously described. The LP-9 cell line is an untransformed, diploid, mesothelial cell line that was derived from a 26-year old female ovarian cancer patient and was obtained from the NIGMS Human Genetic Cell Repository at the Coriell Institute (AG07086) (Camden, NJ). A673 cells were purchased from ATCC (Manassas, VA). UB27, UF5, and UED5 cells with tetracycline-repressible expression of WT1 (-KTS), EWSR1-WT1(-KTS), or EWSR1-WT1(+KTS) have been described [16, 37]. JN and BER cells were grown in 10% FBS DMEM/F12 media and LP9 cells were grown in 15% FBS DMEM/F12 media that were supplemented with 10 ng/mL EGF (ThermoFisher, Waltham, MA) and 0.4 ug/mL hydrocortisone (Sigma-Aldrich, St. Louis, MO). For UB27, UF5, and UED5 cells, 1μg/mL tetracycline was added to suppress expression of WT1 (-KTS), E-KTS or E+KTS. Inducible shRNA stable cell lines were selected using puromycin (0.5μg/mL) and G418 (250μg/mL) (Sigma-Aldrich), and shRNA expression was induced with 1μg/mL doxycycline (dox) (Sigma). A673 cell line have been authenticated by ATCC through short tandem repeat DNA profile and DSRCT cell lines were previously authenticated for the presence of EWSR1-WT1 via qPCR, and were tested for mycoplasma contamination. Cells were transfected using Lipofectamine 3000 or RNAiMAX (Invitrogen, Waltham, MA). Two independent siRNAs targeting *WT1* (SASI_Hs01_00130271, SASI_Hs01_00130272), *SIK1* (SASI_Hs01_00239672, SASI_Hs01_00239673), *SIK2* (SASI_Hs02_00054682, SASI_Hs02_00054683), *SIK3* (SASI_Hs02_00173106, SASI_Hs02_00357920), or siScramble (AM4635) were purchased from ThermoFisher.

### Generation of dox-inducible shRNA cell lines

Dox-inducible LT3-GEPIR vector [38] was modified (see Supplementary Materials for details) and used to generate stable cell lines in JN and BER cells. The shRNA sequences against *WT1* 3′UTR, *SIK1*, or Renilla luciferase listed below were inserted into XhoI and EcoRI sites of the modified vector. Cells were transfected with shWT1, shSIK1, or shRenilla plasmids and selected 48 h post-transfection with puromycin (0.5 μg/mL). All stable cell lines were validated by qRT-PCR and Western blot analyses with or without dox.

shWT1: 5′ GCAGCTAACAATGTCTGGTTA 3′

shSIK1: 5′ GTTCAGCTGATGAAGCTTCTG 3′

Renilla: 5′ AGGAATTATAATGCTTATCTA 3′

### Microarray and RNA-seq analysis

Detailed RNA preparation for microarray and RNA-seq analysis are described in Supplementary Materials. Microarray gene expression profiling was performed with Affymetrix (Santa Clara, CA) Human Genome U133 plus 2.0 Arrays. Three biologic replicates were used for each sample. The data were analyzed using an Affymetrix RMA algorithm. Transcripts with greater than 1.5-fold difference and *P*-value of <0.05 were selected by ANOVA using Partek Pro (Partek, St. Louis, MO). Gene ontology (GO) analysis was performed on RNA-seq data using DAVID v6.7 (The Database for Annotation, Visualization and Integrated Discovery; [39, 40]). RNA-seq libraries were loaded onto Illumina Novaseq 6000 (San Diego, CA) for 75 bp paired end read sequencing. The fastq files were generated using the bcl2fastq software for further analysis. RNA-sequencing reads were pseudoaligned to the human transcriptome GRCh38 using kallisto [41]. Differential transcript expression was calculated using DESeq2 (v1.24.0) and further processed through the lfcShrink function with apeglm (v1.6.0) applied to report absolute magnitude of gene expression [42]. Differentially expressed genes (*p* < 0.05) were analyzed using Ingenuity Pathway Analysis (Qiagen, Hilden, Germany. https://www.qiagenbioinformatics.com/products/ingenuity-pathway-analysis) [43].

### Chromatin Immunoprecipitation (ChIP) and promoter-reporter assays

ChIP assay was adapted and performed as previously described [44] using anti-WT1 (ThermoFisher) or rabbit IgG antibodies. For luciferase reporter assay, JN-DSRCT genomic DNA was extracted by DNAzol Reagent (Invitrogen) and 2 kb SIK1 proximal promoter (P4) was PCR amplified from JN-DSRCT-1 genome then inserted into pGL3Basic firefly luciferase vector (Promega, Madison, WI) using Gibson Assembly Cloning kit (NE BioLabs, Ipswich, MA). Truncated promoter plasmids, P1-P3, were generated by PCR amplification followed by digestion and ligation into NheI and XhoI sites. Primers sequences can be found in Table S5. U2OS cells were seeded in 24-well plates and transfected with 0.5 μg SIK1 promoter constructs and 0.5 μg pcDNA3-E-KTS, pcDNA3-E+KTS or empty pcDNA3 vectors along with 0.1 μg of Renilla Luciferase plasmid using Lipofectamine 3000 (Invitrogen). Luciferase activities were measured at 48 h post-transfection using Dual Luciferase Reporter Assay (Promega).

### Western blot, PCR, and Quantitative real time-PCR (qRT-PCR) analysis

Detailed methods for Western Blot assay and qRT-PCR analysis are described in the Supplementary Materials and Methods. ChIP-PCR and qRT-PCR primers sequences are listed in Supplementary Tables S5 and S6.

### Cell cycle analysis

Dox-inducible shRenilla or shSIK1 stable JN and BER cells were treated with or without 1 μg/mL dox for 48 h (JN) or 24 h (BER) before double thymidine block and for the remaining culture period. Cells grown overnight (~16 h) with 2 mM thymidine media were released with 20% FBS media for 12 h, and were incubated overnight with 2 mM thymidine media again. Cells were released with 20% FBS media, collected at the indicated time points, fixed in 70% ethanol, stained with propidium iodide and subjected to flow cytometry analysis (FACSymphony A3, BD Biosciences, Franklin Lakes, NJ). To quantify DNA replication following SIK1 depletion, cells were treated as above and 5 h post-release into S phase, cells were incubated with 20 μM EdU for one hour. Cells were fixed and stained with propidium iodide and EdU using Click-iT EdU Alexa Fluor 647 Flow Cytometry Assay Kit (C10424, Invitrogen) followed by flow cytometry. Cell-cycle analysis was performed using FlowJo Software (BD Biosciences). Three independent experiments were performed and analyzed for cell cycle and DNA replication analyses.

### Xenografts in immune-deficient mice

All animal procedures were approved by the Tulane Institutional Animal Care and Use Committee. Male NOD-scid-IL2Rγ-null (NSG) mice (6 weeks) were purchased (Jackson Laboratory, Bar Harbor, ME) and used for all xenograft studies. Dox-inducible JN- and BER-shRNA cells were resuspended in PBS and mixed with equal volume of Matrigel (Corning, Tewksbury, MA) and subcutaneously injected into the lower flanks (2 injections/mice) of NSG mice. When tumors were palpable, mice were randomized and placed with normal or dox-containing (0.2% dox/2% sucrose) drinking water (*n* = 6 mice each group). The sample size (*n* = 6) was estimated based on our prior experience with xenografts to achieve significant statistical power. Dox-containing water was replaced every 3 days and tumor volume was measured every 3 days using a digital caliper. Tumor volume was calculated as: length × (width)^2^ × 0.5, where length is the largest diameter and width is perpendicular to the length. Mice were sacrificed at 4 weeks (BER) or 6 weeks (JN) post-dox treatment. All mice were intraperitoneally injected with 50 mg/kg (b.w.) EdU 8 h prior to sacrifice. Tumors were harvested, weighed, fixed in formalin, embedded in paraffin, sectioned (5 μm) and stained with H&E or stained for EdU using Click-iT EdU proliferation kit (Invitrogen) [45]. Double-blind evaluation was used to count Hoechst33342+ cells and EdU+ cells in the images taken at 10× magnification (Nikon Eclipse 80i microscope; NIS-Elements software, Melville, NY) from three random areas per slide. The average percentage of EdU positive cells were calculated for each tumor, with at least *n* = 12 tumors evaluated per treatment group.

### Cytotoxic assays

Cells were seeded in 96-well plates at 1 × 10^4^/well and treated with increasing doses of YKL-05-099 (SelleckChem, Houston, TX), Prexasertib (SelleckChem) or DMSO (control). For combined treatments, cells were treated with 0.5 nM Prexasertib and increasing doses of YKL-05-099. Cell viability was measured with CCK-8 assay (Sigma-Aldrich) at 72 h post-treatment and absorbance was measured using Clariostar microplate reader (BMG Labtech, Cary, NC). All experiments were repeated at least three times in triplicates per each dose.

### Statistical analysis

All experiments included a minimum of three independent replicates. The sample size was chosen based on prior experience with the assays used to ensure adequate statistical power. Data meet the normal distribution and all data are reported as means ± S.E.M. Two-way ANOVA was used to determined tumor volume differences between control and dox-treated group in xenograft study and Student’s T-test was used for the other experiments using GraphPad Prism 7 program (GraphPad Software, San Diego, CA). A *p*-value <0.05 was considered statistically significant.

## Supplementary information

Hartono-Supplementary Materials, Methods, and Figures

Table S3 List of altered genes following EWSWT1 or SIK1 Depletion

## Data Availability

The microarray and RNA-seq datasets generated and analyzed during the current study are available at GEO under the accession number GSE180031 and GSE197254.
