# Supplementary material for: Salt-Inducible Kinase 1 is a potential therapeutic target in Desmoplastic Small Round Cell Tumor
Source: Oncogenesis. 2022 Apr 20;11(1):18. doi: 10.1038/s41389-022-00395-6 (PMC9021191; doi:10.1038/s41389-022-00395-6)
Supplement: Supplementary file 1 — Hartono-Supplementary Materials, Methods, and Figures [file 41389_2022_395_MOESM1_ESM.pdf]

Supplementary Materials for

Salt-Inducible Kinase 1 is a potential therapeutic target in Desmoplastic Small Round Cell Tumor

Alifiani Bonita Hartono[1], Hong-Jun Kang[1], Lawrence Shi[1], Whitney Phipps[1], Nathan Ungerleider[1], Alexandra Giardina[1], WeiPing Chen[2], Lee Spraggon[3], Romel Somwar[3], Krzysztof Moroz[1], David H. Drewry[4], Matthew E. Burow[5], Erik Flemington[1], Marc Ladanyi[3] and Sean Bong Lee[1]

Corresponding author: Sean Bong Lee, Ph.D., Tulane University School of Medicine, Department of Pathology and Laboratory Medicine, 1700 Tulane Ave. Room 808, New Orleans, LA 70112; Tel: (504) 988-1331; Fax: (504) 988-7389; E-mail: slee30@tulane.edu

<u>This PDF file includes:</u>

Supplementary Materials and Methods

Fig. S1: Native WT1 expression in DSRCT and SIK1 CNV and genomic DNA sequencing analyses in DSRCT cell lines

Fig. S2: EWSR1-WT1 directly binds to and regulate SIK1 proximal promoter

Fig. S3: siRNA-mediated depletion of EWSR1-WT1 or SIK1

Fig. S4: SIK1 is essential for DNA replication and MCM2 phosphorylation in DSRCT

Fig. S5: Pan-SIK inhibitor YKL 05-099 inhibits MCM2 phosphorylation in DSRCT

Fig. S6: SIK1 transcript and protein levels in xenograft tumors with or without doxycycline

Fig. S7: Dose-response curves of DSRCT and LP9 cells to Prexasertib

Table S1: List of 201 DSRCT-enriched and 74 DSRCT-repressed genes.

Table S2: List of nonsynonymous changes between SIK1 and SIK1B loci.

Table S3: List of altered genes following EWSR1-WT1 or SIK1 depletion. (Excel file)

Table S4: List of altered genes in IPA “cell cycle control of chromosomal replication” pathway.

Table S5: ChIP-PCR and reporter-promoter assay primers.

Table S6: qPCR and Saenger Sequencing Primers

Table S7: Antibodies used for Western Blot and ChIP

Supplementary Materials and Methods

Dox-inducible shRNA vector modification

The original dox-inducible LT3-GEPIR vector was modified due to leaky expression of GFP that was observed in the absence of doxycycline upon initial transfection. Removing the RSV promoter and the 5' LTR from the original LT3-GEPIR by using SapI digest and re-ligating the vector abrogated doxycycline independent expression of GFP. The modified LT3-GEPIR vector was then used to insert shRNA sequences into the XhoI and EcoRI sites.

Lentivirus production and transduction

To generate EWSR1-WT1 lentiviral vectors, pCDNA-E-KTS-HA and pCDNA-E+KTS-HA [11] were digested with EcoRI, and *EWSR1-WT1* cDNAs were ligated into the EcoRI site of lentiviral vector pCDH-CMV-MCS-EF1α-copGFP (System Biosciences, Palo Alto, CA). Lentiviruses were produced by co-transfecting 293T cells with the pCDH lentiviral vectors and ViraPower lentiviral packaging mix (Invitrogen) using Lipofectamine3000 (Thermofisher Scientific). Viral supernatants were collected 48 hours and 72 hours post-transfection, and concentrated with LentiX-Concentrator (Takara Bio, San Jose, CA) according to manufacturer instructions. LP9 cells were transduced with pCDH-Empty Vector (control), pCDH-E-KTS-HA, or pCDH-E+KTS-HA in the presence of polybrene (8µg/ml) for 16 hours. Cells were selected with puromycin (1µg/ml) at 48h post-transduction. Total RNAs and proteins were isolated 7 days after selection and analyzed as indicated.

Colony Formation Assay

For colony formation assay, dox-inducible cells were seeded at $1x10^4$ cells/well in 6-well plates and grown for 2 weeks in the absence or presence of dox (1µg/mL). Cells were stained with crystal violet (0.5%), washed and dried overnight, then photographed. To quantify stained colonies, 1mL of methanol

was added to each well to extract the dye and optical density was measured at 570 nm with a plate reader. Three independent experiments with biological replicates were performed.

Genomic and RNA-Extraction, and Real-time qPCR assays

Genomic DNA was isolated with DNazol (Thermo Fisher Scientific) according to manufacturer's protocol. To determine CNV, 60ng of gDNA was used as template and qPCR was performed using specific Taqman probes targeting *SIK1* exon 13 (chromosome 21) and *RPP30* (chromosome 10). Total RNAs were isolated with RNA-STAT60 (Tel-Test, Friendswood, TX), and 500ng total RNAs were reverse transcribed into first-strand cDNA (iScript cDNA synthesis kit, Bio-Rad, Hercules, CA). Relative transcript levels were analyzed by real-time qPCR using SYBR Green (SsoAdvanced Universal SYBR Green Supermix, Bio-Rad) and calculated by the comparative Ct method normalized against human β-ACTIN. Primers are listed in Supplementary Table S6.

Immunoblot

Cell lysates were prepared in lysis buffer supplemented with cOmplete EDTA-Free Protease Inhibitor Cocktail (Sigma-Aldrich), 1mM NaF, and 2mM Na_3VO_4. Proteins were resolved in 8% or 10% SDS-PAGE gels and transferred onto a 0.45 um nitrocellulose membrane (Bio-Rad). Membranes were blocked with either 5% non-fat milk or 5% BSA TBST buffer and incubated with primary antibodies at 4C overnight, followed by secondary antibodies LI-COR IRDye 800CW goat anti-Mouse (#926-32210, 1:15,000 dilution) or LI-COR IRDye 680RD goat anti-Rabbit (#926-68071, 1:15,000 dilution) and scanned on LI-COR Odyssey CLx (Lincoln, NE). At least two independent immunoblots were performed for each experiment, with a representative immunoblot shown. Antibodies are listed in Supplementary Table S7.

Microarray and RNA-seq RNA preparation

For Microarray analysis, JN-DSRCT cells were transfected with an shRNA targeting the 3' UTR of WT1 or control (scrambled) and briefly selected for 3 days with 0.5μg/mL puromycin. For RNA-seq analysis, total RNAs were prepared using RNeasy kit (Qiagen, Hilden, Germany). For RNA-seq analysis, the sequencing libraries were constructed from 100-500ng of total RNA using the Illumina TruSeq Stranded Total RNA kit with Ribo-Zero (San Diego, CA) following the manufacturer instruction. The fragment size of RNAseq libraries was verified using the Agilent 2100 Bioanalyzer (Agilent, Santa Clara, CA) and the concentrations were determined using Qubit instrument (ThermoFisher).

Fig. S1

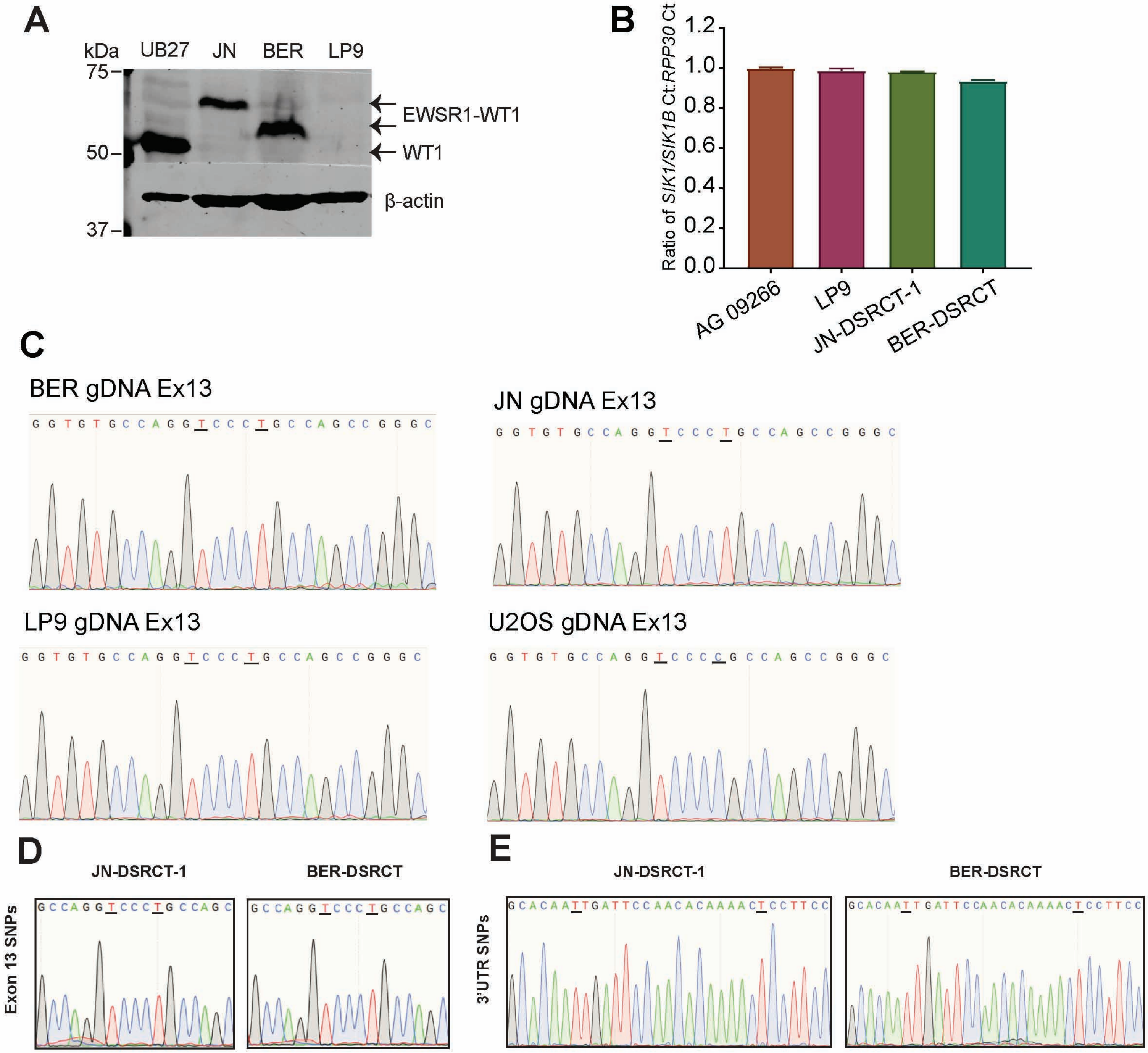

Fig. S1 WT1 is not expressed in DSRCT and SIK1 CNV and genomic DNA sequencing analyses in DSRCT cells. (A) JN-DSRCT-1, BER-DSRCT, and LP9 do not express native WT1 protein. Lysates from JN, BER, LP9 and UB27 cells were immunoblotted with antibodies against the C-terminus of WT1 or β-ACTIN. UB27 is a TET-OFF inducible cell line expressing WT1(-KTS), and served as a positive control. **(B)** TaqMan qPCR analysis of SIK1 and RPP30 with genomic DNAs from DSRCT, LP9 and normal human fibroblast (AG09266). **(C)** Sequencing analysis of SIK1 exon 13 genomic DNA from DSRCT, LP9 and U2OS cells. Note the presence of only SIK1B SNPs (T) and the absence of SIK1 SNPs (C) at the underlined locations, demonstrating that SIK1 and SIK1B genes are not duplicated in any cell lines. Sequencing of RT-PCR products amplifying the exon 13 **(D)** and 3'UTR **(E)** regions of SIK1 and SIKB in JN- and BER-DSRCT cells. Underlined nucleotides represent SNPs from Val615 variant.

Fig. S2

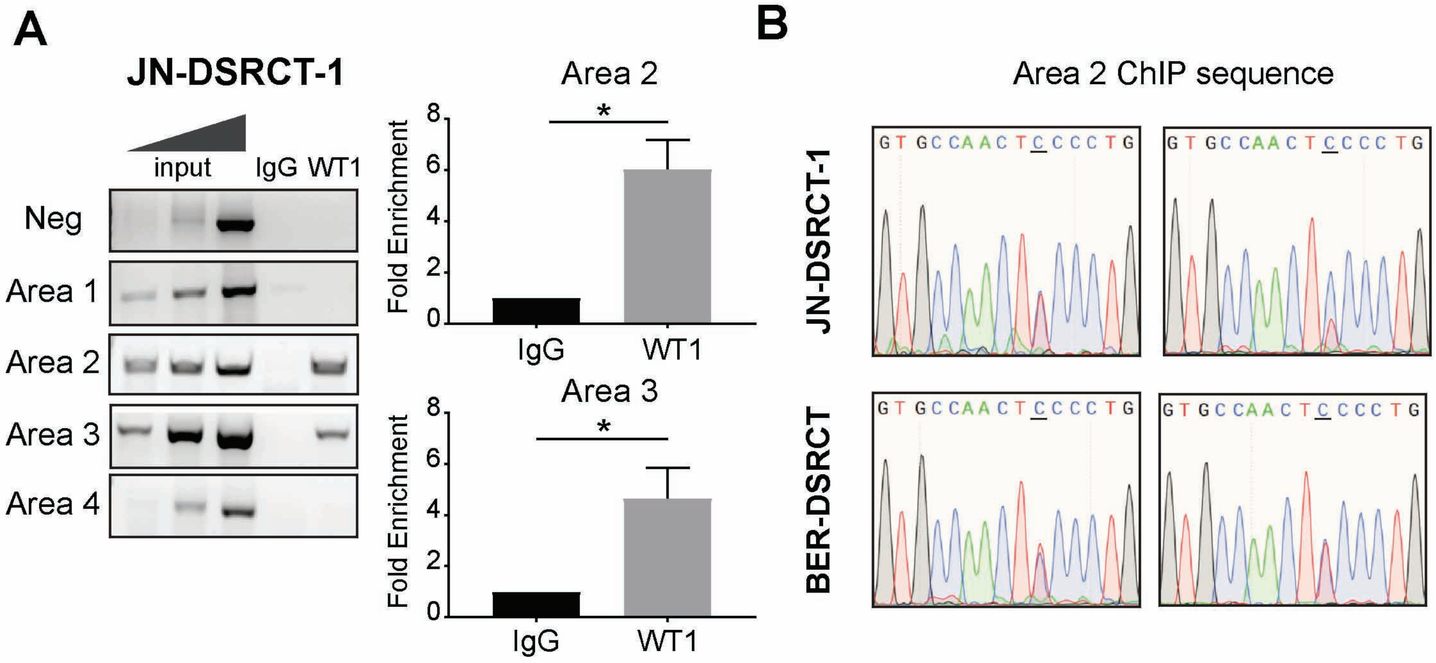

Fig. S2 EWSR1-WT1 directly binds to and regulate SIK1 proximal promoter. (A) JN-DSRCT-1 cells were crosslinked with 1% formaldehyde and DNA was sheared using M220 Covaris sonicator. EWSR1-WT1 was precipitated with anti-WT1 C-term antibody (Thermofisher). Area 1, Area 2, Area 3, Area 4, and negative regions were PCR-amplified with the indicated primers (see Fig1C). Band intensities were quantified using ImageJ and normalized to input (n=3, **$p<0.01$, mean ± SEM, student t-test). **(B)** EWSR-WT1-immunoprecipitated ChIP samples were sequenced using the Area 2 primers (see Fig1C) and underlined nucleotide denotes a SNP in the proximal promoter region. Note that two nucleotides are present (C and T) in the underlined base, demonstrating the presence of both SNPs in *SIK1* promoter regions in the ChIP samples.

Fig. S3

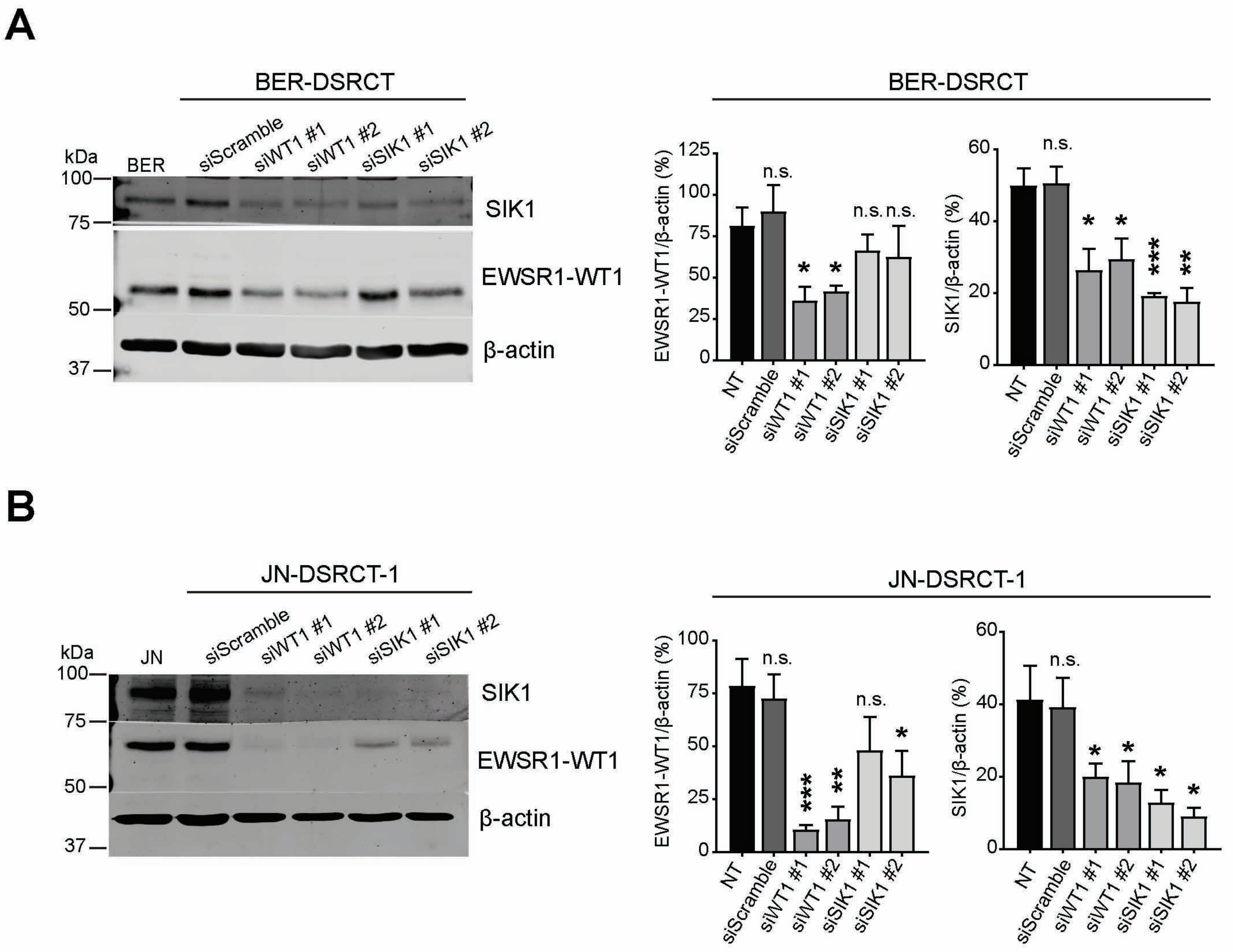

Fig. S3 siRNA-mediated depletion of EWSR1-WT1 or SIK1. Western blot analysis of EWSR1-WT1, SIK1, and β-ACTIN following transient transfection with two independent siRNAs against WT1, SIK1 or a Scrambled control in BER **(A)** and JN **(B)** cells. The right panels show quantification of EWSR1-WT1 and SIK1 normalized to β-ACTIN from four independent Western blot experiments (*p<0.05, **p<0.01, ***p<0.001, mean ± SEM, student t-test).

Fig. S4

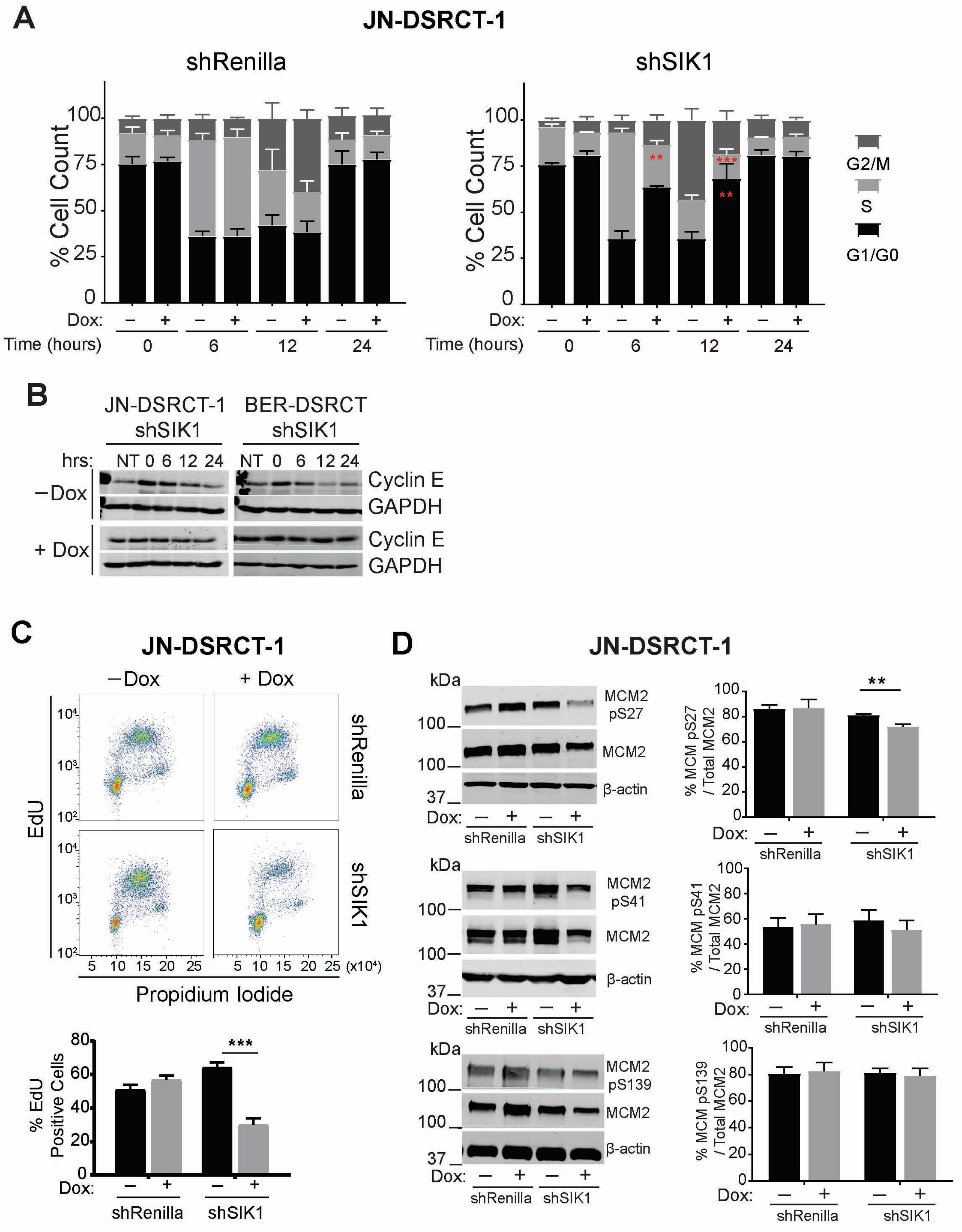
A
JN-DSRCT-1
shRenilla
shSIK1
% Cell Count
G2/M
S
G1/G0
Dox:
Time (hours)
0
6
12
24
B
JN-DSRCT-1 shSIK1
BER-DSRCT shSIK1
hrs: NT 0 6 12 24
−Dox
+ Dox
Cyclin E
GAPDH
C
JN-DSRCT-1
−Dox
+ Dox
shRenilla
shSIK1
EdU
Propidium Iodide
(x10⁴)
% EdU Positive Cells
D
JN-DSRCT-1
kDa
MCM2 pS27
MCM2
β-actin
MCM2 pS41
MCM2 pS139
% MCM pS27 / Total MCM2
% MCM pS41 / Total MCM2
% MCM pS139 / Total MCM2

Fig. S4 SIK1 expression is essential for DNA replication and MCM2 phosphorylation in DSRCT. (A) Cell cycle analysis of G1-arrested shRenilla or shSIK1 DSRCT cell lines with or without Dox treatment following release to S phase. Quantification from three independent experiments are shown below in bar graphs. **(B)** Western blot analysis of Cyclin E levels in G1-arrested shSIK1 JN and BER-DSRCT cells following release to S phase. **(C)** EdU incorporation analysis of G1-arrested shRenilla and shSIK1 JN-DSRCT-1 cells following release to S phase. Five hours post-release, cells were pulsed with EdU [20 μM] for 1h and analyzed by flow cytometry (n=3, *** $p<0.001$, mean ± SEM, student t-test) **(D)** Western blot analysis with phospho-specific antibodies to S27, S41 and S139 of MCM2. MCM2 antibody was used to determine the total levels of MCM2. Phosphorylation signal intensity was normalized to total amount of MCM2 (n=3, ** $p<0.01$, mean ± SEM, student t-test).

Fig. S5

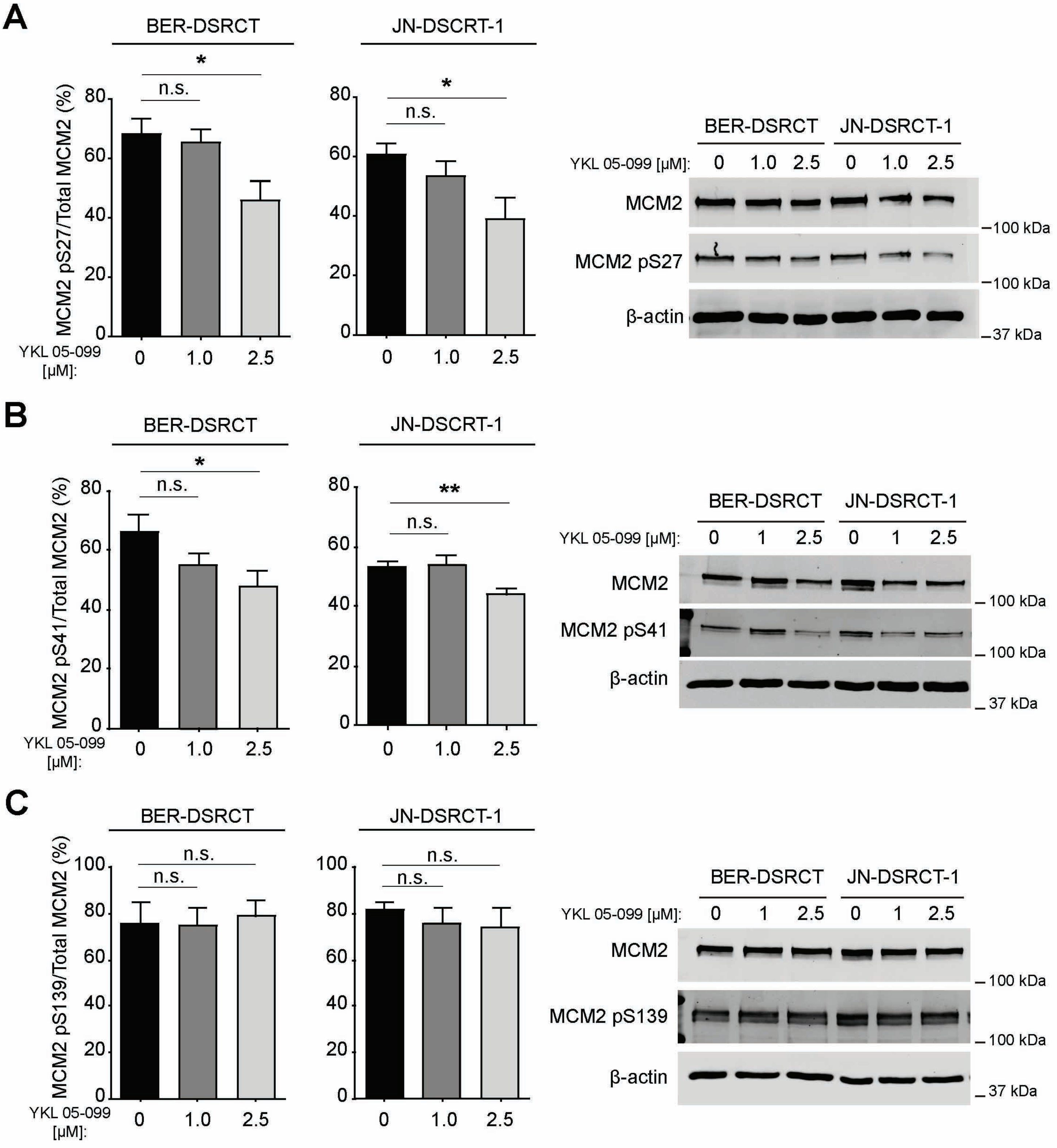

Fig. S5 Pan-SIK inhibitor YKL 05-099 inhibits MCM2 phosphorylation in DSRCT. Western blot analysis with phospho-specific antibodies to **(A)** S27, **(B)** S41 and **(C)** S139 of MCM2 following treatment with YKL-05-099 at indicated doses. MCM2 and β-ACTIN antibodies were used to determine the total levels of MCM2 and as a loading control. Phosphorylation signal intensity was normalized to total amount of MCM2 from four independent experiments and shown as bar graphs (*p<0.05, **p<0.01, mean ± SEM, student t-test).

Fig. S6

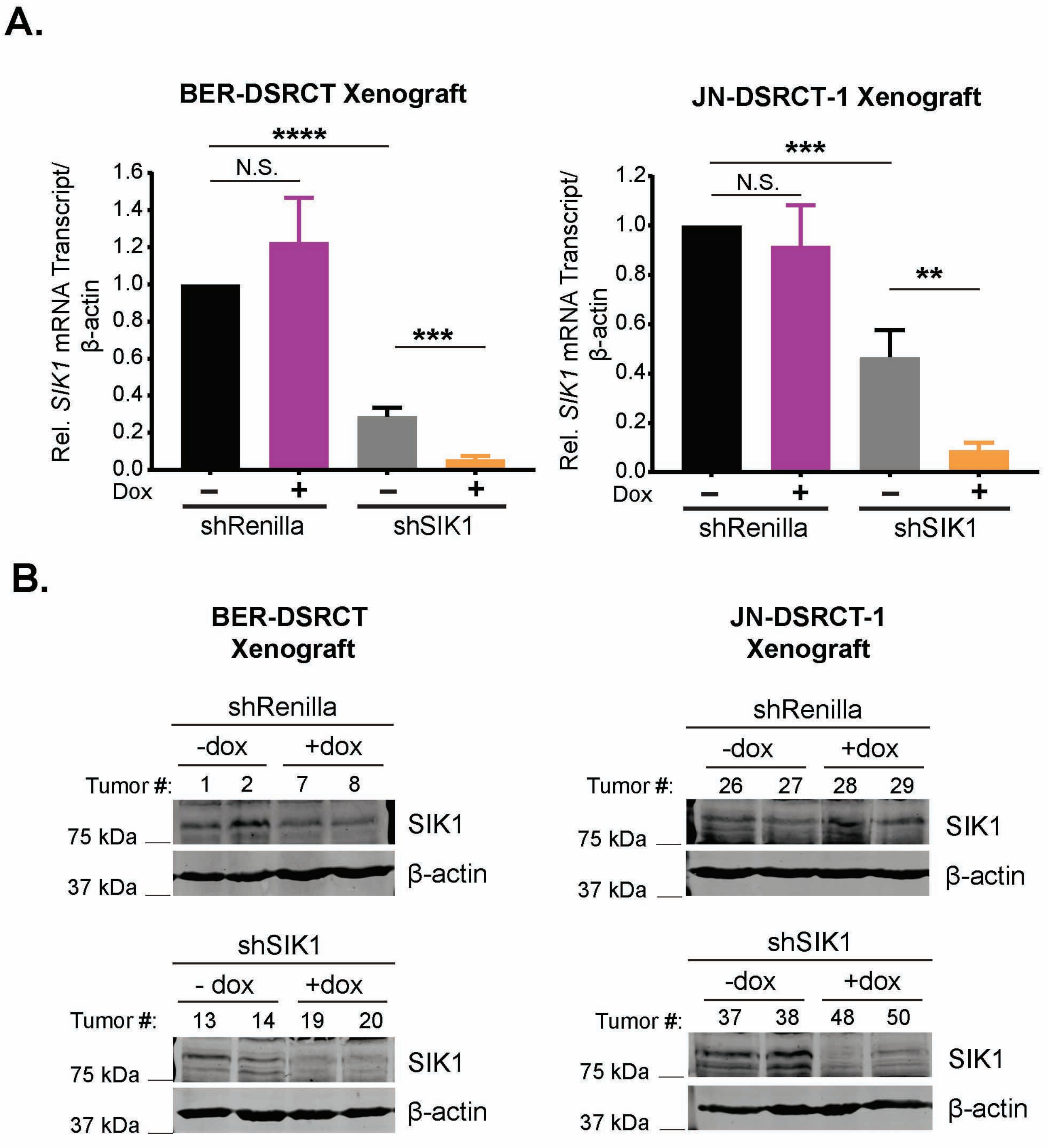

Fig. S6 SIK1 transcript and protein levels in xenograft tumors with or without doxycycline. (A) qRT-PCR analysis of SIK1 transcripts normalized to β-ACTIN from xenograft tumors (n=6, ****p<0.0001, ***p<0.001, mean ± SEM, student *t*-test) and **(B)** Western blot analysis of SIK1 and β-ACTIN from xenograft tumors (n=2).

Fig. S7

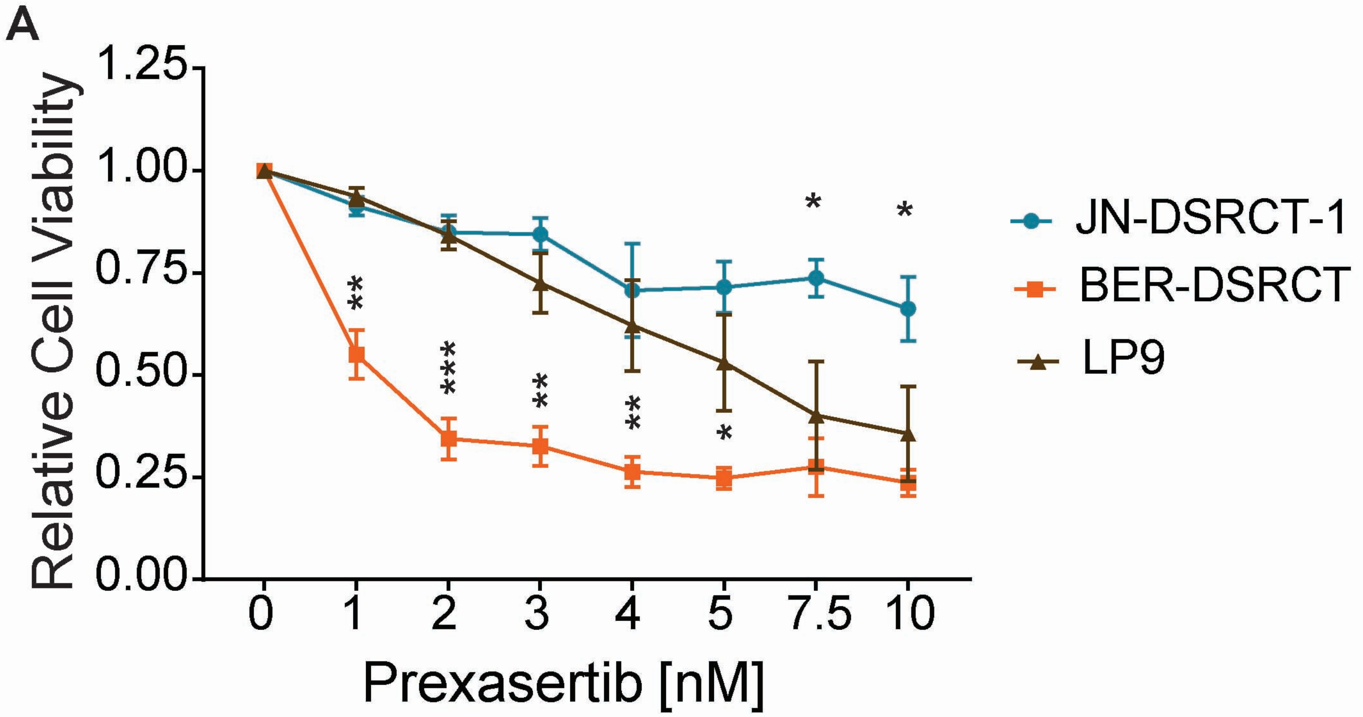

Fig. S7 Dose-response curves of DSRCT and LP9 cells to Prexasertib. JN-DSRCT-1, BER-DSRCT and LP9 cells were treated with increasing doses of prexasertib. DSRCT cell viability values were compared to LP9 cell viability at each dose (*p<0.05, **p<0.01, ***p<0.001, mean ± SEM, student t-test).

Table S1 Integrated gene expression analysis of sarcoma microarray data and EWSR1-WT1 depleted JN-DSRCT-1 cells. List of genes that are enriched or repressed in primary DSRCT vs. other sarcomas (ES, SS, ARMS, ASPS) and upregulated or down regulated following the shRNA mediated knockdown of EWSR1-WT1 in JN-DSRCT-1 cells.

DSRCT-enriched transcripts				DSRCT-repressed transcripts	
ABCB1	CXCR7	KCTD12	SEMA4F	ADK	SCG2
ABCB1 /// ABCB4	CXXC4	KIAA0232	SERPINB1	ALDH6A1	SEL1L3
ABI2	CYP2B6 /// CYP2B7P1	KIF26B	SERPINB9	ANXA1	SLC38A6
ACTB	DACT1	KLF11	SGPL1	ANXA5	SP110
ACTG2	DDX18	KLHL25	SHANK2	ARG2	SPP1
ADARB1	DIAPH1	LARGE	SIK1	ATP1B1	STC1
AES	DIO3	LBH	SLC19A1	C1orf54	STEAP3
AIM1	DNALI1	LCK	SLC26A6	CASP4	STOM
AJAP1	DOK1	LGALS2	SMPDL3B	CCDC28A	TBC1D8
AKAP17A	DOK5	LHX2	SMURF2	CITED2	TMEM135
ALLC	DPF1	LPAR1	SNAI2	CREBL2	TMX4
APOA1	DUSP2	LPCAT4	SNAPC2	CREG1	TOB1
APOD	DUSP6	LPP	SNED1	DBNDD2 /// SYS1 /// SYS1-DBNDD2	TPP1
AQP4	DUSP9	LRRC8E	ST5	DNAJC15	TTN
ARC	EGFL6	LSS	ST6GAL1	EFHC1	TUBA4A
ARHGAP10	EGLN3	LZTS1	ST6GALNAC5	ENPEP	UBR2
ARPP19	EGR1	MAGEL2	STON1	FADS3	UCP2
ARTN	EGR3	MERTK	TCF3	FAM111A	UQCRC2
ASCL1	ELAVL1	MLF1	TFRC	FAM46A	VWA5A
ASMTL	ENTPD6	MLLT10	TGIF2	FDX1	
ASTN1	EPHA1	NACC2	TH	GBP2	
ATXN1	EPHB3	NAP1L2	TLE2	GCHFR	
BAI1	FBXO21	NEDD4L	TMEM2	GTDC1	
BAIAP3	FBXO41	NR4A1	TOMM34	HIST1H1C	
BDKRB2	FGFR4	NR4A3	TP53BP2	KDSR	
C11orf95	FOS	NRXN1	TSPAN7	LHFP	
CA12	FOXA2	NTRK3	TYRO3	LY96	
CACNA1C	FOXD1	PALLD	VAV2	LYN	
CACNA2D2	FOXF1	PARP12	VDR	MAP1A	
CALHM2	GAL	PBX1	WIPF2	MBD2	
CAMK2A	GATA6	PCDH11X /// PCDH11Y	WIZ	MBNL1	
CAMSAP1	GNB3	PCNX	WNT6	ME2	
CAPN2	GPR116	PDE10A	WT1	MID1	
CAPN5	H6PD	PDLIM7	YAP1	MSRA	
CARD10	HEXIM1	PLCL1	ZMYND10	MTO1	
CATSPERB	HIPK2	PLEKHA6	ZYX	N4BP2L2	
CBLN1	HLX	PMF1		NNT	
CCDC40	HTR1E	POMT2		NSMAF	
CCL25	IGF2 /// INS-IGF2	PPP1R12B		PARM1	
CCND1	IL1RAPL1	PRR5L		PCDH17	
CD24	IL2RB	PTCH1		PDE4DIP	
CD9	IL3RA	PTH1R		PDE8A	
CDH4	IL6ST	PXDN		PHKB	
CELSR1	IL8	PYCARD		PIK3R1	
CHI3L1	INSIG2	PYGB		PLK1S1	
CHRFAM7A /// CHRNA7	IPO9	RABGAP1		PNP	
CIRBP	IQCG	RBFOX1		PSME1	
CNN1	IRX4	RBM38		PTEN	
COL13A1	IRX5	RFC5		PTN	
COL18A1	ISG20	RGS12		PTPRG	
COL8A2	ITGA6	RNF24		RAP1A	
CRTC3	ITIH2	ROCK2		RHOQ	
CSGALNACT1	ITIH5	RRAD		RNF6	
CUX1	KCNB1	SAE1		RRAGD	
CX3CL1	KCNQ2	SDC3		SAMD4A	

Table S2. List of nonsynonymous changes between SIK1 and SIK1B loci in ~19kb regions encompassing promoters, exons, introns and 3' UTRs.

	Nucleotide		Position in Chr 21 (GRCh38)	
**Location**	**SIK1**	**SIK1B**	**SIK1**	**SIK1B**
Promoter	T	C	43,437,405	6,105,108
Promoter	T	C	43,431,385	6,106,874
Promoter	-	A[a]	43,431,175[b]	6,107,085
Promoter	C	A	43,430,880	6,107,380
Promoter	-	T[a]	43,428,357[b]	6,109,905
Promoter	-	A[a]	43,428,356[b]	6,109,906
Promoter	G	T	43,428,323	6,109,939
Promoter	C	T	43,428,190	6,110,072
Intron	T	G	43,426,733	6,111,529
Intron	T	C	43,419,773	6,118,489
Exon 13	C	T	43,417,674	6,120,588
Exon 13	C	T	43,417, 670	6,120,592
Intron	T	A	43,417,201	6,121,061
3'UTR	G	A	43,416,332	6,121,930
3'UTR	T	C	43,415,627	6,122,635
3'UTR	C	T	43,415,420	6,122,842
3'UTR	C	T	43,414,944	6,123,318
3'UTR	C	T	43,414,926	6,123,812

Promoter encompasses ~8kb upstream of the transcription start site.

[a] indicates insertion

[b] Corresponding base pair location +1 to the insertion site

Table S4: List of altered genes in IPA cell cycle control of chromosomal pathway. Depletion of EWSR1-WT1 or SIK1 in DSRCT cells changes the expression of genes that are part of IPA cell cycle control chromosomal pathway.

IPA: Cell Cycle Control of Chromosomal Replication

	JN shWT1 Microarray		JN and BER shSIK1 RNA-seq	
**Symbol**	**Expr Fold Change**	**Expr p-value**	**Expr Log Ratio**	**Expr p-value**
CDC45	-3.912	6.88E-04	-1.67	6.69E-06
CDC6	-3.146	7.27E-05	-1.919	2.97E-06
CDK18	1.04	8.57E-01	-0.673	4.24E-03
CDK3	1.063	6.20E-01	1.237	7.96E-04
CDT1	-3.138	1.24E-04	-2.42	4.31E-13
DNA2	-2.197	1.28E-02	-1.228	1.02E-06
LIG1	-1.416	1.80E-03	-1.333	1.41E-05
MCM2	-3.445	2.56E-05	-1.824	2.96E-06
MCM3	-2.996	3.39E-04	-1.333	5.94E-04
MCM4	-3.316	1.74E-04	-1.687	2.06E-04
MCM5	-3.158	5.38E-04	-2.054	1.35E-07
MCM7	-1.787	9.62E-04	-1.512	1.35E-04
MCM9	1.315	4.48E-03	0.881	3.53E-04
ORC1	-2.28	6.27E-04	-2.023	5.47E-08
ORC3	-1.303	5.49E-04	-0.793	1.19E-03
ORC6	-1.856	4.08E-04	-1.621	9.84E-10
POLA1	-1.828	7.45E-03	-0.912	2.08E-04
POLA2	-2.212	3.10E-03	-1.778	1.15E-09
POLD1	-1.605	8.44E-03	-1.514	1.19E-06
POLE	-1.752	7.87E-03	-0.704	5.43E-03
PRIM1	-1.799	2.11E-03	-1.78	1.95E-05
PRIM2	-1.344	4.17E-02	-1.035	7.18E-03
RPA2	-2.012	2.05E-03	-1.605	3.28E-07

Supplementary Table S5: PCR Primers for ChIP-PCR and Promoter-Reporter Assay

**ChIP-PCR**	**Primer Sequence (5'-3')**	
	**Forward**	**Reverse**
Area 1	TTCCAGAGCGGCCGGTCACG	ATGAGGCCGTTGCCCTGGC
Area 2	GGCGATTGGGAAATCTGTGGTCAG	GCCTCATCCGAACGGAAAAC
Area 3	AGGCTCACGCCATCCTCCCAC	CTGACCACAGATTTCCCAATCGCC
Area 4	CTGGGTCAGGGTGAAAGGTCAAG	CTGAGCAAGAGAACAAGGCCC
Negative	TAGGAGTGAGTCCGCGGGGCTCAG	TCGATCCGGATCCCAGGACCGTC
**pGL3-PCR inserts**	**Primer Sequence (5'-3')**	
	**Forward**	**Reverse**
Gibson-PCR1-P4	GGTACCGAGCTCTTACGCGTGCTAGCCCCTGGGTCAGGGTGAAAGGTCAAG	GCCTCATCCGAACGGAAAACGTTTTCTTCAGGGGAGACAG
Gibson-PCR2-P4	CTGTCTCCCCTGAAGAAAACGTTTTCCGTTCGGATGAGGC	TTAGATCGCAGATCTCGAGCCCATGAGGCCGTTGCCCTGGCG
P1	TTTGCTAGCTTCCAGAGCGGCCGGTCACG	TTT CTCGAG ATGAGGCCGTTGCCCTGGCG
P2	TTTGCTAGCGGCGATTGGGAAATCTGTGGTCAG	TTT CTCGAG ATGAGGCCGTTGCCCTGGCG
P3	TTTGCTAGCAGGCTCACGCCATCCTCCCAC	TTT CTCGAG ATGAGGCCGTTGCCCTGGCG

Table S6: qPCR and Saenger Sequencing Primers

Genes	Refseq No.	Primer Sequence (5'-3')		
		**Forward**	**Reverse**	
B-actin	NM_001101.3	GCAAAGACCTGTACGCCAAC	AGTACTTGCGCTCAGGAGGA	
WT1	NM_024426.6	CCATACCAGTGTGACTTCAAGG	TGTGGGTCTTCAGGTGGTC	
SIK1	NM_173354.5	CTTCTCCGCACACAGCTACA	GTTTTGCAGTGACTCCACCG	
SIK2	NM_015191.3	CCCAGAAACGAGAGGTCCAC	TGCTACAATTCCCTGGGTGAG	
SIK3	NM_025164.6	AGCACCGTACCAACCTGATG	ATGTGCACTGTGAGCCTCTG	
				**Probe (5' - 3')**
RPP30 gDNA	NC_000010.11	GATTTGGACCTGCGAGCG	GCGGCTGTCTCCACAAGT	CTGACCTGAAGGCTCT
SIK1 gDNA Ex13	NG_052009.1	TGGAGCCTGCTGGAGGAG	GGAGATAACCTGCCGTGTGC	TAGAGCAGCAGAGGTAGGGC
				**Sequencing primer (5' - 3')**
SIK1 gDNA Ex13 PCR	NG_052009.1	TGTCACCCGTCCTTTCCTTACC	GAACAGAGGACAGGTGGTCACT	CTCGAGGAGATAACCTGCCGTG
SIK1 Ex13 mRNA	NM_173354.5	TCACCGCGCCATGTATAGTC	TCCATCTCACAGTCCCCCTG	GCTTGGGAGGAGCTGTTCTG
SIK1 3'UTR	NM_173354.5	GCTGTTTCAAAGACTGGGCG	GCTATTGCATTGCCGTCTGTATT	CTTGCCGCAGAGAAAACACC

Table S7: Antibodies used for Western Blot and ChIP

Antibodies	Company	Cat#
WT1 C-term	ThermoFisher	PA5-16879
SIK1	Proteintech	51045-1 AP
MCM2	CST	12079
MCM2 pS27	Abcam	109459
MCM2 pS41	Abcam	109270
MCM2 pS139	CST	12958
Cyclin B	CST	4128
β-Actin	Sigma	A4700
GAPDH	CST	5174
Rabbit IgG	CST	2729
