# Supplementary material for: Salt-Inducible Kinase 1 is a potential therapeutic target in Desmoplastic Small Round Cell Tumor
Source: Oncogenesis. 2022 Apr 20;11(1):18. doi: 10.1038/s41389-022-00395-6 (PMC9021191; doi:10.1038/s41389-022-00395-6)
Supplement: Supplementary file 2 — Table S3 List of altered genes following EWSWT1 or SIK1 Depletion [file 41389_2022_395_MOESM2_ESM.pdf]

Table S3: List of altered genes following EWSR1-WT1 or SIK1 depletion

	JN shWT1		JN and BER shSIK1	
Gene	Exp. P-value	Exp. Fold Change	exp. P-value	Exp. Log ratio
KCNC1	6.84E-03	-2.39E+01	2.71E-04	-6.84E-03
PLB1	4.94E-03	-1.20E+01	1.92E-12	-4.94E-03
AGBL1	2.23E-04	-9.36E+00	4.50E-36	-2.23E-04
PTGS1	2.53E-04	-8.94E+00	7.84E-05	-2.53E-04
LCK	3.23E-04	-7.97E+00	3.41E-06	-3.23E-04
SYT2	4.02E-03	-7.86E+00	5.58E-20	-4.02E-03
ST3GAL1	6.38E-05	-7.79E+00	2.05E-02	-6.38E-05
DLX3	5.62E-04	-7.67E+00	2.93E-03	-5.62E-04
BDKRB2	6.91E-04	-6.64E+00	2.44E-18	-6.91E-04
CCL25	9.03E-03	-6.61E+00	3.77E-07	-9.03E-03
CELF4	3.90E-03	-6.51E+00	8.32E-19	-3.90E-03
DEGS2	6.34E-04	-6.14E+00	9.47E-09	-6.34E-04
ARTN	3.03E-03	-6.08E+00	1.93E-19	-3.03E-03
CTCFL	1.09E-02	-5.98E+00	1.25E-10	-1.09E-02
UNC5A	5.04E-03	-5.98E+00	1.10E-22	-5.04E-03
AMHR2	6.27E-04	-5.70E+00	8.44E-03	-6.27E-04
TH	3.11E-03	-5.58E+00	1.71E-04	-3.11E-03
SLC29A4	2.03E-04	-5.50E+00	1.32E-09	-2.03E-04
PLXNA4	1.10E-03	-5.42E+00	1.65E-17	-1.10E-03
LRRC55	1.64E-03	-5.40E+00	2.59E-02	-1.64E-03
KCTD12	2.08E-03	-5.13E+00	3.90E-25	-2.08E-03
PEG3	1.78E-03	-5.05E+00	2.77E-16	-1.78E-03
CXCL1	1.70E-03	-5.02E+00	2.89E-13	-1.70E-03
ACKR3	2.91E-04	-4.97E+00	1.38E-06	-2.91E-04
EXO1	1.59E-04	-4.88E+00	2.76E-04	-1.59E-04
FBXO32	7.14E-03	-4.74E+00	2.17E-22	-7.14E-03
PBK	4.62E-04	-4.71E+00	1.08E-02	-4.62E-04
IL2RB	4.80E-05	-4.66E+00	2.94E-09	-4.80E-05
CXCL8	7.74E-03	-4.62E+00	4.93E-16	-7.74E-03
AURKB	2.63E-04	-4.52E+00	2.43E-05	-2.63E-04
UBE2C	9.65E-04	-4.52E+00	1.61E-02	-9.65E-04
KCNB1	1.33E-02	-4.49E+00	1.15E-14	-1.33E-02
E2F2	4.99E-05	-4.48E+00	5.55E-05	-4.99E-05
FGFR4	5.88E-04	-4.38E+00	1.38E-13	-5.88E-04
RASD2	2.50E-03	-4.37E+00	1.53E-15	-2.50E-03
HIPK2	3.49E-03	-4.36E+00	4.37E-17	-3.49E-03
SNAI2	5.87E-04	-4.34E+00	2.08E-14	-5.87E-04
CACNA2D2	1.15E-03	-4.32E+00	4.73E-16	-1.15E-03
ANO7	1.13E-04	-4.32E+00	1.35E-05	-1.13E-04
MYL7	3.30E-02	-4.23E+00	6.30E-09	-3.30E-02
LOXL3	3.60E-04	-4.20E+00	5.15E-18	-3.60E-04
CA5B	3.97E-03	-4.15E+00	2.55E-05	-3.97E-03
CDC25C	1.60E-03	-4.10E+00	1.05E-04	-1.60E-03
PTH1R	1.40E-02	-4.09E+00	6.30E-03	-1.40E-02
TROAP	1.21E-03	-4.06E+00	6.77E-03	-1.21E-03
BUB1	1.30E-03	-4.02E+00	1.78E-02	-1.30E-03

CIT	3.95E-04	-4.00E+00	2.11E-04	-3.95E-04
ISX	3.06E-02	-3.99E+00	1.50E-14	-3.06E-02
ACTN2	2.06E-04	-3.77E+00	3.80E-08	-2.06E-04
BLM	7.11E-04	-3.76E+00	7.71E-06	-7.11E-04
SLCO4A1	4.92E-04	-3.76E+00	4.52E-05	-4.92E-04
FOXC2	1.82E-03	-3.74E+00	5.22E-07	-1.82E-03
APOA1	3.35E-03	-3.74E+00	3.10E-04	-3.35E-03
NEIL3	2.16E-03	-3.71E+00	4.00E-02	-2.16E-03
SEPTIN3	5.45E-03	-3.70E+00	1.53E-19	-5.45E-03
CAMK2A	1.55E-04	-3.63E+00	1.13E-08	-1.55E-04
B3GNT6	1.07E-03	-3.62E+00	1.78E-20	-1.07E-03
NFAM1	1.12E-04	-3.53E+00	2.33E-08	-1.12E-04
PIF1	6.39E-04	-3.52E+00	1.09E-02	-6.39E-04
ZSCAN5A	8.66E-03	-3.50E+00	3.15E-05	-8.66E-03
KALRN	4.33E-04	-3.49E+00	4.22E-14	-4.33E-04
AP1G2	3.15E-03	-3.46E+00	1.73E-02	-3.15E-03
MCM2	2.56E-05	-3.45E+00	2.96E-06	-2.56E-05
KIFC1	3.43E-04	-3.45E+00	3.83E-04	-3.43E-04
MERTK	3.31E-03	-3.44E+00	4.22E-09	-3.31E-03
FAM20C	2.52E-03	-3.44E+00	2.14E-15	-2.52E-03
SLC8A2	1.97E-04	-3.43E+00	1.21E-05	-1.97E-04
ACP3	2.76E-03	-3.42E+00	4.88E-21	-2.76E-03
RFC5	4.41E-04	-3.41E+00	6.66E-07	-4.41E-04
APOD	3.78E-04	-3.39E+00	2.26E-28	-3.78E-04
ADGRF5	9.82E-05	-3.39E+00	6.28E-03	-9.82E-05
DDX18	8.62E-04	-3.37E+00	4.99E-11	-8.62E-04
BDKRB1	6.89E-04	-3.36E+00	3.02E-08	-6.89E-04
ULBP3	7.30E-04	-3.34E+00	2.35E-02	-7.30E-04
MCM4	1.74E-04	-3.32E+00	2.06E-04	-1.74E-04
RRM2	3.86E-04	-3.31E+00	6.46E-04	-3.86E-04
HTR1E	7.37E-04	-3.28E+00	1.31E-13	-7.37E-04
TCF19	4.49E-04	-3.25E+00	2.25E-04	-4.49E-04
RFX2	3.25E-03	-3.24E+00	1.24E-06	-3.25E-03
POLQ	7.53E-04	-3.23E+00	9.29E-03	-7.53E-04
SNAPC2	3.56E-03	-3.22E+00	8.25E-20	-3.56E-03
BUB1B	1.30E-03	-3.20E+00	1.36E-03	-1.30E-03
LRP8	2.85E-04	-3.19E+00	6.42E-06	-2.85E-04
NTRK3	1.15E-05	-3.19E+00	1.61E-04	-1.15E-05
KCNK9	2.12E-03	-3.18E+00	2.50E-02	-2.12E-03
FOXF1	1.30E-03	-3.17E+00	1.64E-11	-1.30E-03
PPEF1	8.91E-03	-3.17E+00	1.27E-02	-8.91E-03
RAD51	3.64E-05	-3.17E+00	5.68E-06	-3.64E-05
WDHD1	7.23E-04	-3.16E+00	2.08E-04	-7.23E-04
MCM5	5.38E-04	-3.16E+00	1.35E-07	-5.38E-04
RPS6KA5	2.62E-04	-3.15E+00	1.48E-04	-2.62E-04
TMEM63C	7.72E-04	-3.13E+00	4.06E-08	-7.72E-04
ST3GAL3	5.68E-04	-3.13E+00	4.33E-02	-5.68E-04
AURKA	2.51E-04	-3.10E+00	4.54E-02	-2.51E-04
TK1	4.01E-05	-3.09E+00	1.40E-02	-4.01E-05

CX3CL1	7.41E-04	-3.09E+00	6.59E-04	-7.41E-04
KSR2	1.20E-03	-3.08E+00	8.61E-06	-1.20E-03
CDK1	2.36E-03	-3.08E+00	4.90E-02	-2.36E-03
RRAD	8.94E-03	-3.05E+00	6.96E-07	-8.94E-03
CACNA1C	1.19E-02	-3.02E+00	1.78E-32	-1.19E-02
TRIP13	9.70E-05	-3.00E+00	5.74E-07	-9.70E-05
MCM3	3.39E-04	-3.00E+00	5.94E-04	-3.39E-04
SIK1/SIK1B	2.37E-04	-2.98E+00	6.62E-12	-2.37E-04
EGR2	1.01E-03	-2.97E+00	1.48E-02	-1.01E-03
CITED1	4.54E-02	-2.97E+00	6.40E-26	-4.54E-02
SMURF2	2.15E-03	-2.97E+00	1.19E-05	-2.15E-03
NEK2	1.16E-03	-2.94E+00	4.92E-02	-1.16E-03
DIO3	3.23E-03	-2.92E+00	2.19E-11	-3.23E-03
POLE2	9.02E-04	-2.91E+00	3.28E-07	-9.02E-04
TSHZ3	1.69E-03	-2.91E+00	4.45E-12	-1.69E-03
TBXAS1	5.97E-03	-2.88E+00	1.07E-06	-5.97E-03
MLLT10	6.44E-04	-2.85E+00	6.48E-05	-6.44E-04
CCND1	1.56E-03	-2.85E+00	8.15E-13	-1.56E-03
ESPL1	6.64E-04	-2.83E+00	1.63E-05	-6.64E-04
FOXM1	3.24E-04	-2.82E+00	1.94E-03	-3.24E-04
ERCC6L	2.40E-03	-2.81E+00	1.14E-04	-2.40E-03
UHRF1	5.70E-03	-2.81E+00	2.89E-06	-5.70E-03
TTK	1.35E-03	-2.78E+00	1.21E-02	-1.35E-03
MYBL2	2.43E-04	-2.77E+00	1.38E-02	-2.43E-04
WIZ	2.78E-03	-2.77E+00	7.64E-12	-2.78E-03
CSGALNACT1	3.18E-04	-2.76E+00	8.44E-05	-3.18E-04
SLC25A18	1.62E-03	-2.73E+00	7.68E-09	-1.62E-03
RGS12	2.50E-04	-2.71E+00	2.45E-29	-2.50E-04
FEN1	1.60E-03	-2.70E+00	4.00E-05	-1.60E-03
E2F1	1.29E-04	-2.69E+00	1.13E-04	-1.29E-04
RFC3	3.24E-03	-2.69E+00	2.03E-04	-3.24E-03
CHRNA7	3.06E-04	-2.69E+00	2.46E-15	-3.06E-04
CERS4	2.56E-03	-2.68E+00	2.63E-04	-2.56E-03
PDP2	2.50E-03	-2.68E+00	3.83E-20	-2.50E-03
KCNF1	1.07E-03	-2.66E+00	1.60E-03	-1.07E-03
TLE2	6.96E-03	-2.66E+00	5.81E-11	-6.96E-03
BRIP1	8.84E-04	-2.66E+00	3.64E-07	-8.84E-04
CAPN2	6.79E-04	-2.65E+00	2.64E-03	-6.79E-04
NKX3-1	3.05E-03	-2.64E+00	4.06E-04	-3.05E-03
ADRA2C	6.17E-03	-2.63E+00	5.93E-03	-6.17E-03
FOXA2	3.09E-03	-2.63E+00	1.06E-05	-3.09E-03
ADGRB1	1.49E-03	-2.62E+00	2.57E-13	-1.49E-03
ST6GALNAC5	7.58E-05	-2.61E+00	1.01E-08	-7.58E-05
POMT2	6.96E-04	-2.60E+00	1.69E-14	-6.96E-04
PTTG1	2.36E-04	-2.60E+00	1.48E-03	-2.36E-04
ALOX15	1.42E-03	-2.58E+00	4.32E-02	-1.42E-03
HOXB6	3.95E-02	-2.58E+00	6.20E-07	-3.95E-02
PLK1	1.10E-03	-2.57E+00	3.20E-03	-1.10E-03
FGFRL1	2.14E-03	-2.55E+00	3.59E-04	-2.14E-03

SSH1	9.98E-03	-2.55E+00	3.56E-04	-9.98E-03
FBXO5	5.80E-04	-2.54E+00	2.59E-02	-5.80E-04
PKDCC	1.50E-02	-2.51E+00	4.89E-02	-1.50E-02
PTCH1	1.90E-02	-2.49E+00	1.70E-05	-1.90E-02
FOXP4	8.24E-04	-2.46E+00	5.85E-03	-8.24E-04
HSPA5	3.06E-03	-2.45E+00	5.52E-07	-3.06E-03
CCNB1	7.94E-04	-2.45E+00	1.70E-02	-7.94E-04
KIF20B	2.25E-03	-2.45E+00	3.87E-02	-2.25E-03
RAD54L	2.43E-03	-2.44E+00	1.56E-09	-2.43E-03
ISG20	1.37E-02	-2.42E+00	3.75E-04	-1.37E-02
HEXIM1	3.51E-03	-2.42E+00	8.72E-06	-3.51E-03
TNFSF13B	6.81E-03	-2.41E+00	7.24E-06	-6.81E-03
EME1	9.58E-04	-2.41E+00	3.45E-05	-9.58E-04
DUSP9	1.94E-03	-2.41E+00	6.50E-07	-1.94E-03
CA12	6.62E-03	-2.40E+00	1.90E-28	-6.62E-03
HS3ST3B1	3.78E-03	-2.39E+00	3.86E-02	-3.78E-03
POLD3	2.81E-03	-2.38E+00	4.62E-06	-2.81E-03
PKMYT1	1.96E-04	-2.38E+00	2.26E-06	-1.96E-04
PDE6D	1.88E-03	-2.37E+00	1.25E-02	-1.88E-03
ELK1	1.78E-03	-2.36E+00	1.13E-05	-1.78E-03
BRCA1	2.80E-03	-2.36E+00	4.17E-05	-2.80E-03
SLC26A6	1.57E-03	-2.35E+00	1.13E-08	-1.57E-03
EGLN3	7.21E-03	-2.35E+00	4.20E-12	-7.21E-03
PHKA1	6.16E-04	-2.34E+00	6.09E-26	-6.16E-04
DOK1	1.50E-03	-2.34E+00	1.44E-08	-1.50E-03
VEGFA	6.83E-05	-2.32E+00	1.28E-04	-6.83E-05
ST6GAL1	1.93E-04	-2.32E+00	2.64E-02	-1.93E-04
NEURL1	8.82E-05	-2.32E+00	1.53E-04	-8.82E-05
SMPDL3B	4.54E-03	-2.31E+00	1.17E-04	-4.54E-03
CYP2S1	7.70E-04	-2.31E+00	3.68E-10	-7.70E-04
TNS2	2.23E-03	-2.30E+00	2.79E-06	-2.23E-03
ROCK2	5.10E-04	-2.28E+00	2.35E-05	-5.10E-04
VRK1	2.72E-03	-2.28E+00	3.46E-03	-2.72E-03
PPM1E	6.14E-03	-2.27E+00	4.97E-02	-6.14E-03
ATP11A	2.43E-04	-2.26E+00	8.19E-04	-2.43E-04
HASPIN	2.43E-02	-2.26E+00	1.48E-02	-2.43E-02
HPD	1.05E-03	-2.25E+00	1.42E-06	-1.05E-03
HELLS	1.16E-03	-2.25E+00	4.07E-03	-1.16E-03
SPAG5	8.67E-04	-2.25E+00	1.48E-03	-8.67E-04
ADSS2	5.61E-04	-2.24E+00	1.39E-02	-5.61E-04
SPTSSA	1.41E-03	-2.24E+00	3.95E-05	-1.41E-03
DIS3L2	3.30E-03	-2.23E+00	3.61E-03	-3.30E-03
POLA2	3.10E-03	-2.21E+00	1.15E-09	-3.10E-03
MASTL	1.63E-03	-2.21E+00	1.60E-03	-1.63E-03
LITAF	2.16E-03	-2.20E+00	4.84E-02	-2.16E-03
MTMR4	9.75E-04	-2.20E+00	1.31E-08	-9.75E-04
ATP8B3	7.72E-03	-2.20E+00	5.75E-05	-7.72E-03
DNA2	1.28E-02	-2.20E+00	1.02E-06	-1.28E-02
CXCL3	2.43E-03	-2.20E+00	4.40E-08	-2.43E-03

PDSS1	5.70E-03	-2.19E+00	1.24E-07	-5.70E-03
RACGAP1	6.78E-06	-2.18E+00	4.53E-04	-6.78E-06
EOMES	4.53E-03	-2.18E+00	2.61E-03	-4.53E-03
CELSR3	4.33E-04	-2.17E+00	4.62E-04	-4.33E-04
NTN1	2.60E-03	-2.17E+00	6.23E-04	-2.60E-03
PDGFRB	3.89E-03	-2.16E+00	1.86E-04	-3.89E-03
NTSR1	8.97E-03	-2.16E+00	7.78E-26	-8.97E-03
RECQL4	3.10E-03	-2.14E+00	4.60E-04	-3.10E-03
DUSP2	4.78E-03	-2.14E+00	4.29E-09	-4.78E-03
PPP2R2A	4.08E-03	-2.14E+00	2.86E-10	-4.08E-03
ITGAX	1.02E-02	-2.13E+00	7.62E-07	-1.02E-02
TNFRSF13C	3.38E-02	-2.12E+00	3.41E-02	-3.38E-02
KLK10	3.54E-02	-2.12E+00	6.01E-03	-3.54E-02
TCF3	1.20E-03	-2.12E+00	1.30E-02	-1.20E-03
ATAD5	2.91E-03	-2.12E+00	1.83E-03	-2.91E-03
GATA6	2.86E-03	-2.12E+00	6.48E-03	-2.86E-03
MYC	4.29E-03	-2.11E+00	7.25E-10	-4.29E-03
PARP12	3.94E-03	-2.11E+00	1.10E-04	-3.94E-03
LPAR1	5.74E-04	-2.11E+00	1.29E-04	-5.74E-04
GSTM3	6.12E-03	-2.11E+00	2.51E-02	-6.12E-03
DDX11	1.93E-03	-2.11E+00	2.25E-04	-1.93E-03
PLK4	3.22E-02	-2.10E+00	1.75E-02	-3.22E-02
EPHB3	1.82E-02	-2.10E+00	6.16E-06	-1.82E-02
MCM8	1.24E-03	-2.09E+00	1.31E-02	-1.24E-03
IGF2	2.16E-03	-2.07E+00	3.30E-04	-2.16E-03
MELK	3.07E-04	-2.07E+00	2.94E-03	-3.07E-04
ASMTL	5.82E-03	-2.07E+00	9.92E-05	-5.82E-03
TRAIP	3.27E-03	-2.06E+00	1.50E-02	-3.27E-03
NQO2	4.68E-02	-2.05E+00	2.00E-02	-4.68E-02
SLC19A1	2.06E-03	-2.03E+00	3.22E-06	-2.06E-03
E2F8	8.48E-03	-2.03E+00	2.91E-06	-8.48E-03
TMF1	6.63E-06	-2.03E+00	4.78E-03	-6.63E-06
RASA3	7.18E-03	-2.02E+00	2.25E-08	-7.18E-03
DHX33	1.01E-03	-2.02E+00	7.62E-05	-1.01E-03
ATAD2	1.66E-03	-2.02E+00	3.57E-08	-1.66E-03
FOXJ3	6.28E-03	-2.01E+00	4.23E-03	-6.28E-03
TENT5C	4.76E-02	-2.01E+00	1.01E-06	-4.76E-02
HK2	4.90E-03	-2.00E+00	4.46E-05	-4.90E-03
FBXO21	5.63E-03	-2.00E+00	3.76E-02	-5.63E-03
POLR3K	4.29E-03	-1.99E+00	9.08E-03	-4.29E-03
DUSP6	1.34E-02	-1.99E+00	9.79E-07	-1.34E-02
RPP30	3.39E-03	-1.98E+00	7.94E-03	-3.39E-03
APBA1	1.44E-02	-1.97E+00	1.11E-03	-1.44E-02
ZFHX2	9.42E-03	-1.96E+00	4.49E-05	-9.42E-03
CBX2	1.14E-03	-1.96E+00	2.74E-05	-1.14E-03
RTEL1	1.21E-03	-1.95E+00	2.55E-02	-1.21E-03
E2F3	2.69E-03	-1.94E+00	1.64E-03	-2.69E-03
FZD5	3.39E-03	-1.94E+00	6.31E-03	-3.39E-03
DOC2B	1.19E-02	-1.93E+00	1.17E-03	-1.19E-02

PYCARD	1.15E-02	-1.93E+00	9.21E-04	-1.15E-02
UBIAD1	5.86E-03	-1.93E+00	3.96E-04	-5.86E-03
VAV2	9.36E-05	-1.92E+00	3.09E-09	-9.36E-05
VASH1	2.10E-03	-1.92E+00	3.71E-15	-2.10E-03
TFRC	7.03E-03	-1.91E+00	4.79E-10	-7.03E-03
GATA4	3.12E-03	-1.91E+00	4.76E-03	-3.12E-03
XRCC3	1.59E-04	-1.91E+00	1.56E-04	-1.59E-04
ASIC1	6.10E-03	-1.91E+00	2.04E-05	-6.10E-03
IL3RA	2.60E-02	-1.91E+00	6.60E-18	-2.60E-02
AQP4	4.07E-02	-1.91E+00	1.87E-04	-4.07E-02
ZNF93	2.53E-03	-1.90E+00	4.68E-02	-2.53E-03
MGAT3	1.46E-02	-1.90E+00	5.20E-04	-1.46E-02
CBL	6.88E-03	-1.90E+00	1.72E-04	-6.88E-03
CELF3	2.48E-02	-1.89E+00	2.40E-04	-2.48E-02
EBF2	2.30E-03	-1.89E+00	1.15E-03	-2.30E-03
MYRF	3.29E-02	-1.88E+00	2.82E-14	-3.29E-02
NUDT1	1.16E-02	-1.88E+00	4.06E-05	-1.16E-02
GRN	2.33E-02	-1.88E+00	3.00E-05	-2.33E-02
NRXN1	1.14E-03	-1.87E+00	8.14E-10	-1.14E-03
SCUBE1	2.86E-03	-1.87E+00	2.61E-06	-2.86E-03
TLE5	6.41E-05	-1.87E+00	2.46E-04	-6.41E-05
HNRNPD	3.10E-02	-1.86E+00	1.50E-03	-3.10E-02
MTHFD2	9.72E-04	-1.86E+00	1.78E-10	-9.72E-04
FIGNL1	4.83E-03	-1.85E+00	3.10E-02	-4.83E-03
LHX2	6.91E-03	-1.84E+00	2.44E-09	-6.91E-03
VDR	1.97E-04	-1.84E+00	6.00E-10	-1.97E-04
PASK	4.13E-03	-1.83E+00	2.04E-04	-4.13E-03
INTS6	1.29E-02	-1.83E+00	9.98E-07	-1.29E-02
RFWD3	1.89E-03	-1.83E+00	2.17E-03	-1.89E-03
POLA1	7.45E-03	-1.83E+00	2.08E-04	-7.45E-03
XYLB	3.26E-02	-1.83E+00	4.08E-07	-3.26E-02
SELENOI	3.11E-02	-1.83E+00	3.55E-04	-3.11E-02
MYO19	2.21E-04	-1.83E+00	5.26E-03	-2.21E-04
ILF3	5.61E-03	-1.82E+00	1.05E-03	-5.61E-03
PCSK6	2.17E-03	-1.82E+00	7.33E-07	-2.17E-03
DIRAS1	5.09E-03	-1.82E+00	4.85E-03	-5.09E-03
HUNK	1.05E-02	-1.82E+00	3.63E-02	-1.05E-02
TAF4B	1.44E-03	-1.81E+00	2.52E-06	-1.44E-03
PDGFA	1.58E-03	-1.81E+00	4.39E-10	-1.58E-03
CUX1	8.52E-03	-1.80E+00	3.01E-06	-8.52E-03
BARD1	1.82E-02	-1.80E+00	2.86E-02	-1.82E-02
ITPK1	3.80E-03	-1.80E+00	4.99E-02	-3.80E-03
PRIM1	2.11E-03	-1.80E+00	1.95E-05	-2.11E-03
NUDT4	5.14E-04	-1.80E+00	2.76E-17	-5.14E-04
KCNK15	7.17E-03	-1.80E+00	4.38E-12	-7.17E-03
IRX4	5.03E-03	-1.79E+00	2.19E-04	-5.03E-03
USP37	1.83E-02	-1.79E+00	1.22E-04	-1.83E-02
MSH2	3.74E-03	-1.79E+00	2.06E-02	-3.74E-03
MCM7	9.62E-04	-1.79E+00	1.35E-04	-9.62E-04

SHMT2	6.49E-04	-1.79E+00	1.21E-05	-6.49E-04
SMC1A	2.02E-03	-1.78E+00	6.56E-03	-2.02E-03
RRM1	4.49E-03	-1.78E+00	6.25E-03	-4.49E-03
GNB3	2.87E-04	-1.78E+00	1.77E-07	-2.87E-04
SUV39H1	2.74E-03	-1.78E+00	4.95E-05	-2.74E-03
GALNT14	3.91E-02	-1.77E+00	8.54E-10	-3.91E-02
PRKDC	2.02E-03	-1.76E+00	3.11E-07	-2.02E-03
UBE2V2	1.71E-02	-1.76E+00	2.55E-02	-1.71E-02
LSS	6.39E-03	-1.76E+00	1.40E-02	-6.39E-03
NXT2	9.34E-03	-1.76E+00	2.19E-03	-9.34E-03
PPIH	1.47E-04	-1.76E+00	4.78E-03	-1.47E-04
EXOSC2	3.35E-03	-1.76E+00	1.57E-06	-3.35E-03
FER	1.16E-02	-1.76E+00	3.96E-05	-1.16E-02
POLE	7.87E-03	-1.75E+00	5.43E-03	-7.87E-03
HOXB3	1.63E-04	-1.75E+00	1.45E-02	-1.63E-04
CDC7	6.20E-04	-1.75E+00	4.23E-02	-6.20E-04
CDK6	1.13E-03	-1.75E+00	2.49E-02	-1.13E-03
E2F7	1.22E-02	-1.74E+00	2.01E-08	-1.22E-02
TYRO3	4.69E-02	-1.74E+00	2.66E-02	-4.69E-02
OLIG1	1.98E-03	-1.74E+00	2.45E-06	-1.98E-03
PRRX1	5.20E-03	-1.73E+00	1.92E-06	-5.20E-03
CHEK2	9.36E-04	-1.73E+00	2.50E-02	-9.36E-04
NAALAD2	1.69E-02	-1.73E+00	4.01E-07	-1.69E-02
NACC2	3.98E-03	-1.72E+00	3.03E-04	-3.98E-03
UBE2I	4.59E-03	-1.71E+00	4.27E-02	-4.59E-03
CDK2	8.54E-03	-1.71E+00	2.68E-02	-8.54E-03
FGF17	6.76E-03	-1.71E+00	5.50E-04	-6.76E-03
RAB3A	2.78E-02	-1.71E+00	2.04E-03	-2.78E-02
CNR1	1.32E-03	-1.71E+00	1.07E-09	-1.32E-03
DNMT3B	1.33E-03	-1.71E+00	3.87E-06	-1.33E-03
CAD	3.69E-03	-1.70E+00	1.42E-06	-3.69E-03
PDK1	3.22E-02	-1.70E+00	4.32E-08	-3.22E-02
AEBP1	2.62E-02	-1.69E+00	1.38E-04	-2.62E-02
HS6ST1	6.27E-04	-1.69E+00	7.88E-18	-6.27E-04
HLA-DPA1	1.06E-02	-1.68E+00	3.21E-02	-1.06E-02
RARG	2.55E-03	-1.68E+00	1.59E-04	-2.55E-03
SLC26A2	1.21E-02	-1.68E+00	1.17E-03	-1.21E-02
PAOX	1.21E-02	-1.68E+00	2.82E-02	-1.21E-02
ARHGAP45	2.04E-02	-1.67E+00	2.93E-15	-2.04E-02
ALKBH2	5.55E-03	-1.67E+00	1.46E-05	-5.55E-03
CACNA1H	3.23E-03	-1.67E+00	3.59E-03	-3.23E-03
CEMIP2	3.76E-02	-1.67E+00	3.45E-03	-3.76E-02
ILVBL	2.97E-02	-1.66E+00	8.74E-03	-2.97E-02
SORD	9.33E-03	-1.65E+00	2.41E-03	-9.33E-03
CYB561D2	1.46E-02	-1.65E+00	1.74E-04	-1.46E-02
ADARB1	1.60E-03	-1.65E+00	1.92E-09	-1.60E-03
CDC25A	4.74E-04	-1.65E+00	1.33E-04	-4.74E-04
P3H3	1.56E-02	-1.64E+00	8.77E-08	-1.56E-02
ZNF496	9.77E-03	-1.64E+00	6.48E-04	-9.77E-03

WT1	8.22E-04	-1.64E+00	1.14E-06	-8.22E-04
EFTUD2	7.71E-03	-1.64E+00	1.56E-02	-7.71E-03
ZFPM1	2.70E-02	-1.64E+00	3.99E-03	-2.70E-02
DGKG	1.27E-02	-1.64E+00	2.74E-02	-1.27E-02
MBOAT7	1.05E-02	-1.64E+00	3.28E-03	-1.05E-02
KIF1B	3.49E-04	-1.64E+00	6.99E-03	-3.49E-04
TMC6	2.96E-03	-1.64E+00	1.78E-02	-2.96E-03
TRIM71	1.57E-03	-1.63E+00	2.34E-20	-1.57E-03
AK2	9.49E-05	-1.63E+00	3.71E-02	-9.49E-05
NEDD4L	3.11E-02	-1.62E+00	9.32E-04	-3.11E-02
GSPT1	2.08E-02	-1.62E+00	3.96E-03	-2.08E-02
PFKM	2.90E-04	-1.62E+00	6.31E-03	-2.90E-04
PPP2R5E	1.09E-02	-1.62E+00	6.17E-03	-1.09E-02
KCNJ13	1.71E-02	-1.62E+00	1.17E-06	-1.71E-02
ZFP69B	1.35E-02	-1.62E+00	6.08E-03	-1.35E-02
DFFA	1.45E-02	-1.62E+00	1.16E-02	-1.45E-02
GPR157	5.64E-03	-1.62E+00	8.23E-03	-5.64E-03
PMF1/PMF1-BG	7.41E-05	-1.62E+00	4.77E-02	-7.41E-05
DKC1	2.36E-04	-1.61E+00	3.18E-03	-2.36E-04
BCOR	2.52E-02	-1.61E+00	8.75E-07	-2.52E-02
RAD54B	1.30E-03	-1.61E+00	2.62E-04	-1.30E-03
CENPJ	1.65E-02	-1.61E+00	1.67E-02	-1.65E-02
POLD1	8.44E-03	-1.61E+00	1.19E-06	-8.44E-03
ALYREF	5.96E-03	-1.61E+00	4.37E-02	-5.96E-03
APOL1	3.24E-02	-1.60E+00	5.01E-03	-3.24E-02
CHEK1	6.93E-03	-1.60E+00	5.71E-05	-6.93E-03
REXO1	8.36E-03	-1.60E+00	5.37E-07	-8.36E-03
HLX	4.30E-02	-1.60E+00	5.20E-05	-4.30E-02
ETNK2	3.52E-05	-1.60E+00	6.04E-04	-3.52E-05
USP13	3.54E-03	-1.59E+00	4.08E-05	-3.54E-03
EXOSC9	1.30E-03	-1.59E+00	1.52E-03	-1.30E-03
ACVR2B	6.20E-04	-1.59E+00	2.10E-09	-6.20E-04
UHMK1	3.17E-02	-1.59E+00	4.04E-02	-3.17E-02
GRM2	3.85E-03	-1.59E+00	2.16E-03	-3.85E-03
PDK2	3.71E-02	-1.59E+00	4.12E-05	-3.71E-02
ASIC4	1.79E-02	-1.59E+00	2.88E-05	-1.79E-02
GMNN	2.18E-03	-1.59E+00	2.19E-05	-2.18E-03
FANCM	4.11E-03	-1.59E+00	1.47E-02	-4.11E-03
TAF3	1.82E-03	-1.58E+00	1.49E-07	-1.82E-03
UBE2S	1.98E-04	-1.58E+00	8.03E-05	-1.98E-04
RTN4R	2.27E-02	-1.58E+00	2.51E-03	-2.27E-02
EIF5A	2.44E-02	-1.58E+00	3.17E-02	-2.44E-02
CPT1A	1.40E-02	-1.58E+00	3.60E-06	-1.40E-02
ACYP1	2.34E-04	-1.58E+00	1.58E-03	-2.34E-04
ZNF85	1.26E-03	-1.58E+00	2.71E-02	-1.26E-03
LPCAT4	3.99E-02	-1.58E+00	1.04E-03	-3.99E-02
IPO11	1.03E-03	-1.57E+00	3.24E-02	-1.03E-03
CELSR1	5.35E-03	-1.56E+00	7.41E-14	-5.35E-03
HR	2.21E-02	-1.56E+00	2.93E-02	-2.21E-02

TCOF1	4.76E-03	-1.56E+00	2.16E-04	-4.76E-03
LRRC8E	1.26E-02	-1.55E+00	8.46E-04	-1.26E-02
POLR1E	2.38E-02	-1.55E+00	3.38E-03	-2.38E-02
PFAS	1.88E-02	-1.55E+00	4.02E-10	-1.88E-02
CELF1	1.11E-02	-1.55E+00	2.14E-03	-1.11E-02
PPP3CB	1.30E-03	-1.54E+00	2.86E-02	-1.30E-03
DNMT1	2.34E-03	-1.54E+00	1.33E-05	-2.34E-03
PTPRU	7.35E-03	-1.54E+00	8.65E-12	-7.35E-03
DTYMK	5.10E-03	-1.54E+00	1.20E-02	-5.10E-03
PCID2	2.05E-03	-1.53E+00	1.01E-02	-2.05E-03
DDN	1.25E-04	-1.53E+00	2.78E-06	-1.25E-04
ERG	2.05E-02	-1.53E+00	1.11E-16	-2.05E-02
TONSL	1.52E-02	-1.53E+00	4.35E-05	-1.52E-02
MICAL3	1.62E-02	-1.53E+00	2.87E-02	-1.62E-02
PBX1	2.39E-02	-1.53E+00	1.40E-02	-2.39E-02
CYP27B1	6.76E-03	-1.53E+00	1.09E-02	-6.76E-03
FOXN3	2.94E-02	-1.52E+00	4.31E-06	-2.94E-02
HMGB2	2.69E-03	-1.52E+00	2.76E-05	-2.69E-03
MDM4	3.16E-02	-1.52E+00	3.74E-03	-3.16E-02
GNAT3	3.46E-02	-1.52E+00	1.40E-02	-3.46E-02
CHRNA4	3.77E-04	-1.51E+00	1.44E-10	-3.77E-04
PPP3R1	3.31E-02	-1.51E+00	1.48E-03	-3.31E-02
PDE10A	1.05E-02	-1.51E+00	1.13E-03	-1.05E-02
IPO9	1.37E-02	-1.51E+00	2.10E-02	-1.37E-02
PYGB	1.90E-02	-1.50E+00	1.35E-03	-1.90E-02
SEMA4D	2.51E-02	1.50E+00	3.88E-04	-2.51E-02
FDX1	1.67E-02	1.51E+00	8.84E-03	-1.67E-02
CASD1	1.74E-02	1.51E+00	2.91E-06	-1.74E-02
LRP10	4.66E-02	1.51E+00	1.98E-09	-4.66E-02
IRF6	1.16E-02	1.51E+00	3.10E-02	-1.16E-02
RAB43	3.29E-02	1.51E+00	9.35E-06	-3.29E-02
NADSYN1	1.13E-02	1.52E+00	1.63E-05	-1.13E-02
DTX4	6.47E-04	1.52E+00	6.47E-12	-6.47E-04
ZDHHC3	6.59E-04	1.52E+00	2.57E-06	-6.59E-04
MINDY1	2.21E-03	1.52E+00	2.96E-02	-2.21E-03
PARP4	2.05E-03	1.52E+00	2.37E-02	-2.05E-03
PLCL2	1.47E-03	1.52E+00	4.60E-04	-1.47E-03
CPQ	5.85E-03	1.52E+00	3.22E-04	-5.85E-03
TRIM16	2.37E-02	1.52E+00	3.23E-19	-2.37E-02
TCN2	4.86E-02	1.53E+00	6.46E-16	-4.86E-02
NDST2	1.91E-02	1.53E+00	9.66E-05	-1.91E-02
NFKBIZ	3.92E-02	1.53E+00	5.51E-09	-3.92E-02
SLC25A29	1.31E-02	1.53E+00	8.39E-06	-1.31E-02
NR2F1	6.68E-03	1.53E+00	5.30E-05	-6.68E-03
SIRT7	1.29E-03	1.53E+00	2.09E-02	-1.29E-03
MTHFD2L	1.80E-02	1.53E+00	1.48E-02	-1.80E-02
ICAM1	7.47E-03	1.54E+00	1.93E-09	-7.47E-03
LIFR	7.38E-03	1.54E+00	6.24E-03	-7.38E-03
LRRC8B	4.34E-03	1.54E+00	1.37E-02	-4.34E-03

GNS	2.77E-02	1.54E+00	1.60E-07	-2.77E-02
MME	2.57E-02	1.54E+00	2.54E-05	-2.57E-02
SCD5	2.70E-02	1.54E+00	1.82E-17	-2.70E-02
TTLL7	4.76E-02	1.54E+00	2.55E-02	-4.76E-02
ZC3H12A	1.79E-02	1.55E+00	1.46E-03	-1.79E-02
EXT1	2.25E-02	1.55E+00	9.77E-05	-2.25E-02
OSCP1	5.80E-03	1.55E+00	2.09E-02	-5.80E-03
ABHD11	1.26E-02	1.56E+00	7.70E-19	-1.26E-02
PPP3CA	7.08E-03	1.56E+00	2.15E-17	-7.08E-03
PDXK	1.45E-03	1.56E+00	1.46E-07	-1.45E-03
PAWR	2.06E-03	1.56E+00	1.64E-03	-2.06E-03
RNF6	7.54E-03	1.56E+00	2.40E-02	-7.54E-03
MBD2	2.35E-04	1.57E+00	6.34E-07	-2.35E-04
FZD6	4.75E-02	1.57E+00	3.10E-05	-4.75E-02
EXOSC4	4.78E-02	1.57E+00	2.60E-02	-4.78E-02
STAT1	1.20E-02	1.57E+00	4.20E-12	-1.20E-02
TMEM129	7.74E-03	1.57E+00	3.27E-03	-7.74E-03
TAP1	2.86E-02	1.57E+00	7.53E-14	-2.86E-02
DLG1	1.60E-02	1.57E+00	1.11E-02	-1.60E-02
NEIL1	7.90E-03	1.57E+00	3.81E-05	-7.90E-03
SCG2	2.04E-02	1.58E+00	1.79E-02	-2.04E-02
EXOC7	4.43E-02	1.58E+00	1.57E-05	-4.43E-02
NT5C2	2.20E-03	1.58E+00	1.91E-08	-2.20E-03
ABHD12	4.21E-03	1.58E+00	2.84E-02	-4.21E-03
SIDT2	3.83E-02	1.59E+00	1.23E-02	-3.83E-02
NCOA6	3.11E-02	1.59E+00	4.92E-03	-3.11E-02
PBXIP1	4.29E-02	1.59E+00	7.74E-04	-4.29E-02
PLCE1	1.38E-02	1.60E+00	9.38E-06	-1.38E-02
PTPN14	4.67E-03	1.60E+00	2.70E-02	-4.67E-03
DDX58	6.76E-03	1.60E+00	1.81E-06	-6.76E-03
SLC37A1	5.08E-03	1.60E+00	1.07E-15	-5.08E-03
P2RX4	1.98E-02	1.60E+00	3.25E-02	-1.98E-02
SLC15A2	8.44E-03	1.61E+00	5.02E-03	-8.44E-03
MLLT3	4.61E-02	1.61E+00	3.18E-02	-4.61E-02
APOL6	3.28E-02	1.61E+00	5.34E-05	-3.28E-02
NQO1	1.42E-03	1.61E+00	1.09E-09	-1.42E-03
SLC39A10	1.26E-02	1.62E+00	1.41E-06	-1.26E-02
PGM2L1	2.81E-02	1.62E+00	9.53E-05	-2.81E-02
GALNT6	1.70E-02	1.62E+00	6.71E-04	-1.70E-02
ID4	2.39E-02	1.62E+00	6.78E-13	-2.39E-02
ABCB5	4.45E-02	1.62E+00	4.63E-10	-4.45E-02
GLS2	1.09E-03	1.62E+00	4.12E-05	-1.09E-03
KCNK1	4.29E-02	1.62E+00	3.49E-07	-4.29E-02
ARL4A	7.66E-03	1.63E+00	7.47E-10	-7.66E-03
SPRED3	2.15E-02	1.63E+00	1.20E-02	-2.15E-02
RSPO3	2.41E-02	1.63E+00	2.98E-26	-2.41E-02
GRK3	1.53E-02	1.64E+00	1.86E-13	-1.53E-02
GPR143	9.62E-03	1.64E+00	4.96E-06	-9.62E-03
ZFP36L2	8.92E-03	1.64E+00	2.79E-21	-8.92E-03

SSH3	5.49E-04	1.64E+00	5.13E-07	-5.49E-04
LDB1	3.27E-03	1.64E+00	1.31E-02	-3.27E-03
SLC41A3	1.56E-03	1.65E+00	1.24E-02	-1.56E-03
MIER3	1.78E-02	1.65E+00	3.09E-02	-1.78E-02
F2RL1	1.87E-02	1.65E+00	8.72E-04	-1.87E-02
PDE5A	5.83E-03	1.65E+00	2.15E-03	-5.83E-03
AOPEP	6.07E-03	1.66E+00	5.17E-05	-6.07E-03
AGBL5	3.03E-02	1.66E+00	1.62E-03	-3.03E-02
LATS2	8.68E-03	1.66E+00	2.35E-02	-8.68E-03
PPP1R16B	3.04E-02	1.66E+00	3.31E-20	-3.04E-02
SLC9A7	3.96E-02	1.67E+00	2.50E-06	-3.96E-02
TBX2	1.04E-02	1.67E+00	2.87E-06	-1.04E-02
ZNF20	1.25E-05	1.67E+00	8.74E-03	-1.25E-05
MICB	3.23E-02	1.68E+00	2.78E-02	-3.23E-02
ATP6V0E2	7.03E-04	1.68E+00	1.88E-03	-7.03E-04
SLC2A11	3.69E-02	1.68E+00	2.01E-02	-3.69E-02
PDGFRL	2.00E-02	1.68E+00	3.34E-03	-2.00E-02
TLE1	1.91E-03	1.68E+00	9.04E-09	-1.91E-03
FBXO25	2.40E-04	1.69E+00	1.03E-03	-2.40E-04
SYTL4	6.37E-03	1.69E+00	1.05E-04	-6.37E-03
TJP2	2.33E-04	1.69E+00	3.30E-03	-2.33E-04
DDIT3	4.84E-02	1.69E+00	1.20E-04	-4.84E-02
CTSL	1.95E-02	1.69E+00	8.04E-05	-1.95E-02
GPR158	3.76E-03	1.70E+00	6.07E-03	-3.76E-03
MAN2A1	4.22E-02	1.70E+00	4.51E-09	-4.22E-02
ASL	4.35E-03	1.70E+00	2.31E-09	-4.35E-03
TRMT9B	2.84E-02	1.71E+00	8.42E-07	-2.84E-02
PEAK1	3.61E-02	1.71E+00	1.48E-02	-3.61E-02
SERINC2	3.93E-04	1.72E+00	2.28E-09	-3.93E-04
PIK3R3	4.91E-04	1.72E+00	4.84E-12	-4.91E-04
RGS3	5.21E-03	1.72E+00	9.42E-03	-5.21E-03
PITPNM1	1.34E-03	1.72E+00	4.71E-02	-1.34E-03
MICAL2	7.84E-03	1.72E+00	1.53E-05	-7.84E-03
AKAP12	2.54E-02	1.72E+00	3.02E-10	-2.54E-02
PTPRG	1.30E-02	1.72E+00	1.49E-05	-1.30E-02
SLC4A8	6.77E-03	1.73E+00	6.25E-06	-6.77E-03
FUCA1	2.31E-02	1.73E+00	2.88E-03	-2.31E-02
AMACR	2.31E-02	1.73E+00	5.64E-04	-2.31E-02
SQOR	1.70E-02	1.73E+00	9.92E-03	-1.70E-02
GDE1	3.18E-03	1.74E+00	1.12E-04	-3.18E-03
UEVLD	5.09E-04	1.74E+00	1.38E-02	-5.09E-04
TRIM62	6.17E-04	1.74E+00	3.66E-02	-6.17E-04
ESR1	1.00E-02	1.74E+00	4.26E-09	-1.00E-02
ELF1	1.29E-02	1.74E+00	1.74E-05	-1.29E-02
LRCH4	1.64E-02	1.74E+00	5.01E-05	-1.64E-02
MAP3K8	2.90E-02	1.75E+00	1.72E-02	-2.90E-02
NCOA1	1.11E-03	1.75E+00	1.05E-05	-1.11E-03
CTBS	1.44E-03	1.75E+00	3.20E-02	-1.44E-03
FIG4	1.72E-03	1.75E+00	3.24E-05	-1.72E-03

PIK3R1	4.69E-03	1.76E+00	1.42E-02	-4.69E-03
ASXL1	3.29E-03	1.76E+00	2.92E-02	-3.29E-03
GNG12	3.83E-04	1.76E+00	3.36E-03	-3.83E-04
JAG1	3.38E-02	1.77E+00	1.15E-19	-3.38E-02
NPR1	4.16E-02	1.78E+00	2.76E-06	-4.16E-02
PDE4D	2.49E-02	1.78E+00	5.69E-17	-2.49E-02
CAST	7.80E-04	1.78E+00	5.29E-05	-7.80E-04
KANK1	3.71E-03	1.78E+00	3.78E-04	-3.71E-03
ACOX1	2.68E-03	1.79E+00	1.62E-02	-2.68E-03
LPIN2	2.80E-04	1.79E+00	2.05E-02	-2.80E-04
NAA16	4.00E-02	1.79E+00	8.50E-06	-4.00E-02
CTSS	2.45E-02	1.79E+00	5.42E-04	-2.45E-02
IDS	4.80E-04	1.80E+00	5.88E-06	-4.80E-04
ZNF114	6.93E-03	1.80E+00	2.01E-05	-6.93E-03
NFIB	8.19E-04	1.80E+00	4.56E-02	-8.19E-04
LPAR6	1.51E-02	1.80E+00	1.35E-10	-1.51E-02
DISP1	6.85E-03	1.81E+00	9.96E-15	-6.85E-03
CBLB	1.01E-03	1.81E+00	1.90E-06	-1.01E-03
SOCS3	5.06E-03	1.82E+00	1.20E-04	-5.06E-03
NTF4	2.49E-02	1.83E+00	2.21E-10	-2.49E-02
C11orf54	2.38E-03	1.84E+00	2.96E-03	-2.38E-03
ATP6V1G3	2.69E-03	1.84E+00	2.16E-46	-2.69E-03
LYN	2.02E-03	1.85E+00	1.14E-04	-2.02E-03
SGK1	5.56E-03	1.85E+00	6.39E-22	-5.56E-03
PLD1	6.25E-03	1.85E+00	8.98E-04	-6.25E-03
MARCHF2	6.25E-06	1.85E+00	5.23E-07	-6.25E-06
S1PR3	1.22E-03	1.86E+00	5.93E-08	-1.22E-03
CPD	1.61E-02	1.86E+00	3.32E-04	-1.61E-02
IRAK4	1.24E-03	1.86E+00	1.17E-02	-1.24E-03
BACE1	2.32E-02	1.86E+00	5.05E-05	-2.32E-02
FBXO2	4.83E-02	1.88E+00	4.65E-04	-4.83E-02
RGPD4 (include	4.13E-03	1.89E+00	1.22E-17	-4.13E-03
ZFX	5.67E-03	1.90E+00	1.25E-08	-5.67E-03
CELSR2	1.02E-03	1.90E+00	9.47E-03	-1.02E-03
MAML2	1.05E-02	1.91E+00	5.89E-03	-1.05E-02
ETV7	3.18E-02	1.91E+00	9.19E-03	-3.18E-02
LACTB	2.26E-04	1.93E+00	2.29E-04	-2.26E-04
ISL1	2.68E-04	1.93E+00	8.76E-03	-2.68E-04
PLXND1	1.18E-02	1.94E+00	3.77E-04	-1.18E-02
IL23A	4.18E-02	1.94E+00	1.45E-06	-4.18E-02
RIT1	7.46E-03	1.95E+00	3.60E-03	-7.46E-03
JAK2	8.55E-03	1.95E+00	7.65E-03	-8.55E-03
LIMD1	1.88E-02	1.95E+00	2.51E-05	-1.88E-02
RIMKLA	3.42E-02	1.95E+00	1.84E-03	-3.42E-02
AKT3	4.41E-03	1.95E+00	1.65E-04	-4.41E-03
MAGI1	3.32E-03	1.97E+00	8.47E-07	-3.32E-03
FBXO3	4.11E-02	1.97E+00	1.37E-02	-4.11E-02
IL13RA1	8.84E-03	1.97E+00	1.92E-08	-8.84E-03
SAMD4A	9.94E-03	1.98E+00	5.01E-07	-9.94E-03

CAPN3	7.66E-03	1.98E+00	2.16E-05	-7.66E-03
PANX2	3.14E-03	1.98E+00	3.63E-02	-3.14E-03
SLC16A6	2.97E-02	1.98E+00	8.76E-03	-2.97E-02
IGFBP7	4.75E-02	1.99E+00	7.38E-04	-4.75E-02
IMMP2L	6.37E-06	1.99E+00	6.04E-18	-6.37E-06
SH3RF2	2.27E-02	1.99E+00	1.50E-04	-2.27E-02
ARSB	2.64E-02	2.00E+00	6.06E-03	-2.64E-02
PLAAT3	2.02E-02	2.01E+00	9.62E-04	-2.02E-02
TCP10L	1.48E-02	2.01E+00	1.05E-02	-1.48E-02
SLC41A2	3.88E-02	2.02E+00	2.09E-02	-3.88E-02
ANKRD6	7.36E-03	2.02E+00	1.42E-03	-7.36E-03
ITPR2	1.25E-03	2.03E+00	1.60E-15	-1.25E-03
NCOA7	2.06E-02	2.04E+00	1.78E-03	-2.06E-02
KTN1	4.08E-03	2.05E+00	2.00E-08	-4.08E-03
NRXN3	1.48E-02	2.07E+00	2.93E-04	-1.48E-02
ABCC3	2.75E-02	2.08E+00	1.21E-02	-2.75E-02
NCOA3	6.88E-03	2.08E+00	2.34E-07	-6.88E-03
SLFN5	3.24E-03	2.08E+00	1.04E-06	-3.24E-03
TRPS1	1.05E-03	2.09E+00	3.04E-02	-1.05E-03
SLC1A1	1.55E-02	2.10E+00	6.00E-06	-1.55E-02
CHST13	1.32E-02	2.10E+00	1.86E-02	-1.32E-02
PDE4B	5.33E-03	2.11E+00	4.95E-03	-5.33E-03
ST8SIA4	2.84E-03	2.12E+00	8.30E-05	-2.84E-03
RAB30	4.52E-02	2.12E+00	1.50E-21	-4.52E-02
EIF5A2	4.30E-04	2.13E+00	2.01E-04	-4.30E-04
SLC2A1	3.57E-03	2.13E+00	3.13E-02	-3.57E-03
ABHD4	3.04E-03	2.14E+00	3.61E-04	-3.04E-03
UBR4	2.29E-02	2.15E+00	4.80E-03	-2.29E-02
FAAH2	9.84E-03	2.15E+00	1.45E-04	-9.84E-03
CASP4	2.60E-02	2.16E+00	3.96E-15	-2.60E-02
PCMTD1	2.10E-02	2.16E+00	4.20E-04	-2.10E-02
ANXA1	6.85E-05	2.17E+00	9.68E-13	-6.85E-05
MYLIP	1.71E-03	2.18E+00	2.80E-14	-1.71E-03
LMO4	2.70E-03	2.18E+00	1.21E-07	-2.70E-03
RAB26	4.12E-03	2.18E+00	1.34E-06	-4.12E-03
SPR	9.12E-04	2.18E+00	2.59E-03	-9.12E-04
ACOX2	1.21E-02	2.19E+00	4.75E-02	-1.21E-02
ZBTB20	2.01E-02	2.19E+00	1.02E-03	-2.01E-02
ST14	3.44E-03	2.19E+00	1.17E-13	-3.44E-03
PLCH1	2.74E-02	2.19E+00	5.69E-06	-2.74E-02
DDX60	3.70E-02	2.20E+00	2.11E-02	-3.70E-02
ARID5B	2.22E-05	2.21E+00	2.07E-13	-2.22E-05
SLC4A4	3.03E-02	2.25E+00	1.36E-03	-3.03E-02
SGMS1	1.58E-03	2.25E+00	1.48E-09	-1.58E-03
SOX4	9.54E-04	2.25E+00	1.25E-07	-9.54E-04
PLXNA2	3.23E-02	2.26E+00	1.33E-08	-3.23E-02
IL17D	8.92E-04	2.26E+00	2.17E-02	-8.92E-04
B3GNT2	9.08E-03	2.26E+00	1.49E-05	-9.08E-03
RHOBTB2	4.28E-03	2.27E+00	5.21E-09	-4.28E-03

RNASET2	2.08E-03	2.28E+00	2.97E-09	-2.08E-03
EIF2AK3	7.16E-03	2.28E+00	3.24E-07	-7.16E-03
SLC44A3	1.32E-03	2.29E+00	5.53E-08	-1.32E-03
ANKRD1	6.61E-03	2.30E+00	2.70E-08	-6.61E-03
P4HTM	2.17E-03	2.31E+00	1.50E-05	-2.17E-03
ELF3	4.94E-02	2.32E+00	7.28E-49	-4.94E-02
RAB25	2.03E-03	2.33E+00	5.04E-24	-2.03E-03
ADAM9	2.25E-02	2.34E+00	1.94E-05	-2.25E-02
WDFY3	5.22E-03	2.34E+00	4.72E-05	-5.22E-03
NR0B1	3.93E-02	2.34E+00	4.10E-04	-3.93E-02
GPCPD1	5.81E-04	2.34E+00	2.74E-03	-5.81E-04
MYEF2	7.38E-03	2.36E+00	5.54E-04	-7.38E-03
TRPM8	1.66E-02	2.37E+00	3.86E-05	-1.66E-02
TGM2	1.61E-02	2.37E+00	2.49E-10	-1.61E-02
MTARC1	4.68E-02	2.37E+00	1.02E-02	-4.68E-02
SLC39A8	1.46E-02	2.37E+00	1.68E-10	-1.46E-02
CDS1	2.20E-02	2.39E+00	1.10E-07	-2.20E-02
PON2	6.12E-03	2.40E+00	3.66E-06	-6.12E-03
CEACAM1	1.53E-02	2.41E+00	3.12E-09	-1.53E-02
PINK1	7.48E-04	2.43E+00	1.96E-04	-7.48E-04
TCF4	7.73E-04	2.45E+00	2.57E-07	-7.73E-04
SLC40A1	4.74E-04	2.45E+00	4.18E-10	-4.74E-04
PLAT	1.59E-02	2.46E+00	1.33E-03	-1.59E-02
PRSS8	1.31E-04	2.47E+00	9.48E-13	-1.31E-04
HDAC9	1.36E-03	2.48E+00	1.07E-02	-1.36E-03
SNCA	4.02E-04	2.48E+00	1.96E-04	-4.02E-04
DLL1	1.29E-03	2.48E+00	5.36E-11	-1.29E-03
RGS5	1.03E-03	2.48E+00	8.74E-13	-1.03E-03
AK9	6.58E-03	2.49E+00	1.00E-02	-6.58E-03
ZMIZ2	3.76E-04	2.50E+00	2.35E-03	-3.76E-04
CAMK2D	1.64E-03	2.50E+00	5.52E-04	-1.64E-03
SP110	3.06E-03	2.50E+00	7.79E-03	-3.06E-03
GPR87	6.51E-05	2.51E+00	8.80E-03	-6.51E-05
NEK11	1.37E-03	2.52E+00	8.06E-04	-1.37E-03
ARHGAP5	7.56E-03	2.52E+00	8.41E-05	-7.56E-03
EFNB2	1.49E-02	2.53E+00	1.24E-09	-1.49E-02
PRSS23	3.40E-02	2.54E+00	2.41E-17	-3.40E-02
FOLR1	1.32E-02	2.54E+00	6.37E-06	-1.32E-02
PDGFC	6.95E-03	2.54E+00	1.38E-08	-6.95E-03
VIPR1	2.16E-03	2.56E+00	3.37E-11	-2.16E-03
ERBB3	1.78E-03	2.56E+00	1.05E-27	-1.78E-03
C1GALT1	3.13E-03	2.57E+00	1.14E-04	-3.13E-03
ZSCAN16	8.29E-05	2.57E+00	1.08E-05	-8.29E-05
KLK7	2.59E-02	2.58E+00	5.63E-03	-2.59E-02
FOXO4	4.25E-03	2.58E+00	3.35E-05	-4.25E-03
ACADSB	2.62E-03	2.59E+00	1.74E-05	-2.62E-03
GLCE	8.79E-03	2.59E+00	3.41E-08	-8.79E-03
ANKEF1	6.13E-03	2.60E+00	2.71E-02	-6.13E-03
HES6	2.06E-03	2.61E+00	2.02E-04	-2.06E-03

TNFRSF19	5.20E-04	2.61E+00	2.42E-20	-5.20E-04
IRS2	5.62E-04	2.62E+00	6.15E-05	-5.62E-04
GRHL1	4.48E-03	2.63E+00	3.25E-11	-4.48E-03
SGPP2	3.23E-03	2.65E+00	3.61E-02	-3.23E-03
CMTM8	5.74E-03	2.65E+00	2.38E-10	-5.74E-03
ARL4C	2.79E-03	2.67E+00	4.53E-39	-2.79E-03
NRP1	3.68E-03	2.69E+00	1.15E-31	-3.68E-03
SSBP2	7.37E-03	2.69E+00	5.67E-04	-7.37E-03
P2RY1	2.56E-02	2.70E+00	7.04E-06	-2.56E-02
DUSP16	2.41E-03	2.70E+00	3.95E-09	-2.41E-03
PDE4DIP	5.40E-03	2.72E+00	3.69E-02	-5.40E-03
KLF5	4.62E-03	2.72E+00	4.44E-15	-4.62E-03
ZNF117	6.58E-04	2.73E+00	3.68E-05	-6.58E-04
ANXA9	3.49E-02	2.75E+00	2.47E-09	-3.49E-02
TOM1L2	1.71E-04	2.76E+00	5.25E-07	-1.71E-04
UNC5CL	1.49E-03	2.76E+00	7.80E-11	-1.49E-03
DIO2	1.38E-03	2.77E+00	2.93E-05	-1.38E-03
PROC	6.04E-03	2.79E+00	3.41E-05	-6.04E-03
SLC12A2	1.20E-02	2.81E+00	5.35E-15	-1.20E-02
SCARA3	2.34E-02	2.82E+00	4.72E-09	-2.34E-02
SAT1	6.28E-03	2.84E+00	1.48E-19	-6.28E-03
DTX3L	5.70E-03	2.84E+00	7.35E-07	-5.70E-03
ELOVL7	1.40E-02	2.85E+00	5.58E-15	-1.40E-02
PIK3C2A	2.91E-03	2.85E+00	1.98E-02	-2.91E-03
ID3	1.87E-03	2.93E+00	1.55E-06	-1.87E-03
IL1RAP	3.86E-02	2.94E+00	4.16E-02	-3.86E-02
SP100	3.74E-04	2.96E+00	5.28E-04	-3.74E-04
GLS	2.94E-04	2.97E+00	6.29E-07	-2.94E-04
HES1	3.45E-02	3.01E+00	4.41E-09	-3.45E-02
LYST	2.22E-03	3.03E+00	2.00E-03	-2.22E-03
CITED2	4.23E-03	3.03E+00	4.78E-05	-4.23E-03
SPP1	9.50E-03	3.03E+00	3.97E-08	-9.50E-03
ENPP5	7.13E-03	3.07E+00	5.60E-08	-7.13E-03
PPIC	1.50E-02	3.07E+00	3.75E-05	-1.50E-02
STK17B	1.64E-04	3.10E+00	2.61E-15	-1.64E-04
CAVIN1	1.27E-04	3.11E+00	2.39E-13	-1.27E-04
RUNX2	6.15E-03	3.14E+00	3.62E-02	-6.15E-03
ZDHHC14	2.20E-03	3.14E+00	2.27E-06	-2.20E-03
GPX3	2.03E-03	3.14E+00	1.33E-02	-2.03E-03
PCSK2	1.44E-04	3.17E+00	7.09E-14	-1.44E-04
PXK	6.00E-04	3.18E+00	3.11E-09	-6.00E-04
PGPEP1	1.28E-03	3.20E+00	2.81E-02	-1.28E-03
BMP5	4.32E-02	3.21E+00	1.10E-13	-4.32E-02
SLC2A12	1.48E-02	3.26E+00	3.84E-15	-1.48E-02
STAT4	1.28E-03	3.26E+00	4.20E-02	-1.28E-03
NRG1	2.09E-03	3.28E+00	6.71E-11	-2.09E-03
UCP2	3.15E-04	3.29E+00	2.58E-04	-3.15E-04
MKX	8.58E-04	3.33E+00	3.19E-02	-8.58E-04
CYP26B1	5.25E-04	3.34E+00	3.63E-05	-5.25E-04

GBP2	2.51E-02	3.35E+00	3.56E-07	-2.51E-02
LGALS3BP	4.08E-03	3.39E+00	2.32E-29	-4.08E-03
CFB	1.47E-02	3.40E+00	9.45E-07	-1.47E-02
CPA5	9.61E-04	3.43E+00	6.18E-06	-9.61E-04
AKR1C3	1.14E-03	3.43E+00	3.71E-03	-1.14E-03
ABCA12	5.59E-03	3.53E+00	2.03E-11	-5.59E-03
ETS2	5.15E-05	3.57E+00	1.86E-07	-5.15E-05
PRKACB	1.63E-04	3.62E+00	1.70E-07	-1.63E-04
ADAM28	1.50E-03	3.65E+00	3.65E-13	-1.50E-03
SNCAIP	2.80E-05	3.71E+00	4.45E-19	-2.80E-05
CYGB	1.02E-02	3.84E+00	5.04E-04	-1.02E-02
PARP14	3.51E-02	3.86E+00	1.19E-22	-3.51E-02
VLDLR	4.80E-04	3.89E+00	1.20E-03	-4.80E-04
LGR4	1.02E-03	3.93E+00	1.26E-06	-1.02E-03
GLIS3	2.60E-03	4.13E+00	1.35E-10	-2.60E-03
ADGRL3	2.65E-04	4.16E+00	5.11E-04	-2.65E-04
OXTR	8.56E-04	4.17E+00	4.43E-03	-8.56E-04
NRP2	1.85E-02	4.20E+00	8.00E-05	-1.85E-02
AKR1C1/AKR1C	6.46E-04	4.25E+00	1.73E-15	-6.46E-04
DUSP13	1.01E-04	4.26E+00	1.32E-09	-1.01E-04
F3	1.42E-03	4.28E+00	2.08E-05	-1.42E-03
WNT4	1.16E-04	4.40E+00	1.91E-12	-1.16E-04
RAB40B	8.98E-05	4.46E+00	6.31E-05	-8.98E-05
RAB3B	7.32E-03	4.48E+00	2.37E-03	-7.32E-03
CD274	7.93E-03	4.48E+00	1.82E-06	-7.93E-03
NR5A2	1.24E-04	4.55E+00	1.72E-04	-1.24E-04
CEBPD	4.86E-05	4.71E+00	1.61E-20	-4.86E-05
PARP9	1.78E-03	4.85E+00	4.37E-11	-1.78E-03
CLIC5	5.63E-04	4.87E+00	7.99E-05	-5.63E-04
LIPH	4.47E-04	4.95E+00	1.16E-04	-4.47E-04
EPHA4	2.10E-04	5.05E+00	4.32E-10	-2.10E-04
RND3	9.72E-06	5.07E+00	2.98E-16	-9.72E-06
IFITM1	3.29E-04	5.19E+00	1.31E-05	-3.29E-04
GRHL3	5.22E-04	5.21E+00	1.02E-09	-5.22E-04
ERBB4	4.18E-03	5.27E+00	1.11E-06	-4.18E-03
SOSTDC1	1.33E-03	5.27E+00	8.54E-90	-1.33E-03
CBX5	5.75E-04	5.32E+00	1.82E-05	-5.75E-04
PDP1	1.26E-04	5.35E+00	2.23E-13	-1.26E-04
MYO10	2.06E-04	5.38E+00	2.92E-03	-2.06E-04
CREG1	6.46E-03	5.51E+00	6.88E-23	-6.46E-03
RAB27B	2.45E-04	5.64E+00	1.42E-03	-2.45E-04
MOXD1	6.97E-04	5.74E+00	2.48E-10	-6.97E-04
LARGE2	7.86E-04	5.98E+00	1.91E-08	-7.86E-04
ITGA2	2.87E-03	5.98E+00	1.04E-06	-2.87E-03
CFI	6.62E-04	6.65E+00	5.71E-06	-6.62E-04
ST3GAL6	1.71E-03	6.68E+00	2.04E-04	-1.71E-03
TPK1	2.17E-04	6.73E+00	2.39E-05	-2.17E-04
CDKN2B	1.16E-05	6.77E+00	6.15E-05	-1.16E-05
TM7SF2	1.32E-04	6.87E+00	4.02E-17	-1.32E-04

ABCA5	2.20E-03	6.92E+00	1.02E-08	-2.20E-03
PPBP	5.89E-05	7.27E+00	2.61E-11	-5.89E-05
TGFB2	2.64E-04	7.36E+00	1.60E-03	-2.64E-04
STC1	2.30E-03	7.47E+00	3.77E-13	-2.30E-03
ID1	4.59E-04	7.89E+00	4.12E-31	-4.59E-04
SLC26A7	8.31E-04	8.24E+00	1.93E-05	-8.31E-04
TGFBR2	4.87E-04	8.48E+00	1.84E-28	-4.87E-04
GPRC5B	4.12E-03	8.79E+00	3.39E-60	-4.12E-03
ADGRG6	6.33E-04	8.80E+00	8.17E-09	-6.33E-04
MYO5B	4.11E-04	9.05E+00	6.95E-04	-4.11E-04
FGFR2	1.15E-03	9.84E+00	8.31E-83	-1.15E-03
SCNN1A	4.21E-04	1.03E+01	7.89E-05	-4.21E-04
GDA	1.88E-03	1.04E+01	5.59E-04	-1.88E-03
CHST1	6.57E-05	1.05E+01	4.07E-47	-6.57E-05
ADAMTS6	2.21E-03	1.17E+01	9.42E-16	-2.21E-03
CYP1A1	5.95E-05	1.30E+01	8.85E-05	-5.95E-05
ST6GALNAC1	3.30E-04	1.30E+01	1.53E-19	-3.30E-04
DNM3	7.39E-04	1.31E+01	4.41E-06	-7.39E-04
CP	1.13E-04	1.36E+01	6.06E-31	-1.13E-04
IGF1	1.78E-05	1.41E+01	7.53E-11	-1.78E-05
GPRC5A	8.59E-04	1.41E+01	1.49E-84	-8.59E-04
